# Integrated kinetic, isotherm, ANN, and RSM-based optimization modelling of acid brown 14 removal using ethylenediamine-decorated *Delonix regia* pod biochar

**DOI:** 10.1038/s41598-026-62931-3

**Published:** 2026-08-11

**Authors:** Mohamed A. Hassaan, Mohamed A. El-Nemr, Tarek Othman Said, Oğuzhan Der, Murat Yılmaz, Ahmed Eleryan, Ahmed El Nemr

**Affiliations:** 1 National Institute of Oceanography and Fisheries (NIOF), Elanfoushy, Kayet Bey, Alexandria, Egypt; 2 Department of Chemical Engineering, Faculty of Engineering, Minia University, Minia, 61519 Egypt; 3 The Higher Canal Institute of Engineering and Technology, Al Salam 1-Abu Bakr Al Siddiq Street, Suez, Egypt; 4 Department of Marine Engineering, Maritime Faculty, Bandirma Onyedi Eylul University, Balikesir, Turkey; 5 Department of Chemistry and Chemical Processing Technologies, Bahçe Vocational School, Osmaniye Korkut Ata University, 80000 Osmaniye, Turkey

**Keywords:** *Delonix regia*, Biochar, Ethylenediamine, Adsorption, Acid brown 14 dye, Hydrothermal, Chemistry, Engineering, Environmental sciences, Materials science

## Abstract

The increasing discharge of recalcitrant azo dyes from textile and industrial effluents poses serious ecological and human health risks, necessitating the development of sustainable and cost-effective treatment strategies. The objective of the present study is to examine the adsorption performance of ethylenediamine-functionalized *Delonix regia* pod biochar (DRPB-ED) for the efficient removal of Acid Brown (AB14) 14 dye from aqueous solutions. The approach integrates the valorization of agricultural waste with surface amine functionalization to enhance adsorption performance. Batch experiments demonstrated a maximum removal efficiency of 98.6% under optimized conditions (pH 4.0, 1.0 g/L dosage, 100 mg/L initial concentration, 60 min). Equilibrium data were best described by the Langmuir model, with a maximum monolayer adsorption capacity (*q*_max_) of 60.61 mg/g, indicating favorable monolayer adsorption. Kinetic analysis followed a pseudo-second-order model (R^2^ = 0.99), suggesting dominant chemisorption interactions facilitated by amine functional groups. Process optimization using response surface methodology (RSM) achieved high desirability (D = 0.97), while artificial neural network (ANN) modeling demonstrated strong predictive capability (*R*^2^ = 0.98). Unlike many conventional biochar-based adsorbents that rely solely on physical adsorption, the present study introduces targeted amine functionalization combined with integrated statistical and machine learning modeling, providing both enhanced adsorption performance and robust process predictability. The findings highlight the dual environmental and technological significance of transforming low-cost biomass into high-efficiency adsorbents for sustainable wastewater treatment applications.

## Introduction

 Water contamination has become a pressing global environmental concern, largely driven by increasing industrial activities and urban expansion. Industrial effluents, particularly from textile, paper, and dyeing industries, contribute substantially to water contamination by releasing various synthetic dyes^1^. The discharge of untreated dye effluents is a significant contribution to water contamination. Industrial effluents, particularly from textile, paper, and dyeing industries, contribute substantially to water contamination by releasing various synthetic dyes, plant materials, and aquatic organisms. As a result, dyes, which are discharged by a variety of industrial sectors such as leather, textiles, paint, cosmetics, pulp, and paper, are among the most common contaminants in freshwater. Dyes can affect the aesthetics of aquatic bodies even at low quantities^2,3^. Non-biodegradable dyes adversely affect aquatic ecosystems by decreasing dissolved oxygen levels in water bodies and also pose risks to terrestrial and atmospheric environments when released or utilized through various processes^4,5^. Among these, azo dyes such as Acid Brown 14 (AB14) pose significant ecological and health risks due to their persistence, toxicity, and carcinogenicity. These dyes not only degrade water quality by inhibiting photosynthesis in aquatic systems but also pose serious health hazards to both humans and animals upon exposure^6–9^. Consequently, effective and economically feasible methods for removing these pollutants from water are urgently required^10^. Adsorption has attracted significant attention as an effective and environmentally sustainable method for removing dyes from wastewater^11^. Unlike other treatment processes, such as chemical oxidation, electrocoagulation, and biological treatments, adsorption offers high removal efficiency, operational simplicity, and the potential for reusability of the adsorbent material^12^. Activated carbon has traditionally been employed as an adsorbent owing to its extensive surface area and superior adsorption capacity. Nevertheless, the high production costs of activated carbon limit its large-scale application, spurring interest in alternative, low-cost adsorbents derived from biomass^13^. Biochar, a carbonaceous material produced via biomass pyrolysis, has emerged as an economical and sustainable adsorbent for water treatment applications^14^. Various biomass sources, encompassing agricultural residues and plant-derived materials, have been explored to produce biochars with unique surface properties that enhance their adsorption capabilities^15^. Recent studies highlight the potential of modified biochar composites for targeted dye adsorption applications. Modifications, such as nitrogen doping or the incorporation of functional groups through chemical treatment, can significantly improve the pore structure, surface area, and availability of active sites in biochar, thereby enhancing its adsorption performance^16^. Recent studies have demonstrated that biomass-derived activated carbons combined with statistical optimization tools such as response surface methodology (RSM) can significantly enhance pollutant removal efficiency^17^. Additionally, nitrogen doping and surface functionalization strategies have been shown to improve adsorption capacity and model predictive power^18^. Polymer- and amine-modified carbon materials have also shown enhanced affinity for anionic pollutants, driven by improved electrostatic interactions and surface chemistry^19^. Furthermore, the development of multifunctional carbon-based adsorbents capable of removing both gaseous and aqueous pollutants has attracted growing attention^20^.

Despite these advances, several research gaps remain. First, while *Delonix regia* pod-derived biochars have been explored as adsorbents^21^, their performance has predominantly relied on intrinsic surface properties without targeted molecular-level functionalization. Second, although amine-modified carbons have demonstrated enhanced adsorption performance, limited studies have systematically evaluated ethylenediamine-functionalized *Delonix regia* biochar specifically for azo dye removal. Third, many adsorption studies rely solely on equilibrium and kinetic modeling without integrating statistical optimization and machine learning-based predictive tools, thereby limiting process scalability and predictive robustness.

Delonix regia (Flamboyant tree) pods, an abundant and readily available biomass source, have been investigated in recent studies as a precursor material for biochar production^21^. The high carbon content and structural properties of Delonix regia biochar make it a viable candidate for adsorbing various pollutants. To further enhance its dye-removal efficiency, Delonix regia biochar has been surface-modified with ethylenediamine (ED). Ethylenediamine, which contains amino groups, provides additional adsorption sites via electrostatic interactions and hydrogen bonding, making it particularly effective for adsorbing anionic dyes such as AB14. However, the combined evaluation of adsorption performance, statistical optimization, and artificial intelligence-based predictive modeling for ED-functionalized *Delonix regia* biochar has not yet been comprehensively reported.

Techniques like coagulation/flocculation and adsorption/biosorption have been reported to effectively remove these dyes from both single and binary aqueous solutions^22^, catalytic oxidation^23^, ultrafiltration^24^, ozonation^25–28^, biological activation^29^, microbial fuel cells^30^, and the electro-Fenton process^21–33^, prior to the discharge of synthetic dyes, including DB86 and AY36 dyes, from industrial effluents into the environment^34^.

The present study assesses the removal performance of Acid Brown 14 dye from water using *Delonix regia* pod biochar functionalized with ethylenediamine (DRPB-ED). A systematic investigation of the adsorption process was conducted by assessing the influence of key parameters, including dye concentration, contact time, pH, and adsorbent dosage. To elucidate the adsorption mechanism, various isotherm models—including Langmuir (LIM), Freundlich (FIM), and Temkin (TIM)—were utilized, while kinetic behavior was characterized using models such as pseudo-first-order (PFOM), pseudo-second-order (PSOM), intraparticle diffusion (IPDM), and film diffusion (FDM) models. This study’s results offer important insights into the potential application of DRPB-ED as a low-cost, effective adsorbent for environmental remediation, particularly for treating dye-laden wastewater. The overarching objective is to introduce a sustainable, innovative approach to industrial wastewater treatment to reduce the adverse impacts of azo dye pollution.

Overall, this work aims to valorize agricultural biomass into a functionalized, high-performance adsorbent while offering an integrated modeling strategy for sustainable control of azo dye pollution in industrial wastewater treatment.

## Materials and methods

### Instrumentation and materials

Raw material DRPs were obtained from a local street in Alexandria, Egypt, and used as precursors for the synthesis of the adsorbent DRPB-ED. Sulfuric acid (H_2_SO_4_, 98% purity) was procured from Sigma-Aldrich. The concentration of AB14 dye (Aldrich, USA) was determined by UV–Vis spectrophotometry using a Jena SPEKOL 1300 instrument with 1 cm glass cuvettes. Mixing procedures were performed using a JSOS-500 shaker, and pH measurements were conducted with a JENCO 6173 pH meter. The adsorption–desorption isotherms of DRPB-ED were recorded under a nitrogen atmosphere. Surface characteristics, including specific surface area, pore size, and pore size distribution, were analyzed utilizing a BELSORP Mini II instrument (BEL Japan, Inc.). Adsorption–desorption isotherm modeling was employed to evaluate the monolayer volume (*Vm*, cm^3^ STP), BET surface area (*S*_BET_, m^2^/g), average pore diameter (MPD, nm), total pore volume (*p*_o_/*p*_o_, cm^3^/g), and energy constant (*C*) for DRPB-ED. The Barrett–Joyner–Halenda (BJH) method was applied to determine the micro- and mesoporous surface areas and corresponding pore volumes of DRPB-ED. These calculations were performed with the BELSORP analysis software. Additionally, the BJH method was applied to the desorption isotherm to determine the pore-size distribution. Scanning electron microscopy (SEM; QUALITY 250) was employed to investigate the surface features of DRPB-ED. FTIR analysis of the biochar surface was performed using a VERTEX 70 spectrometer coupled with a V-100 ATR unit, collecting spectra from 400 to 4000 cm^–1^. An SDT650 simultaneous thermal analyzer was used to investigate the thermal properties of DRPB-ED at a constant heating rate of 10 °C min^–1^ from 50 to 1000 °C.

### DRPB-ED preparation

**Fig. 1:**
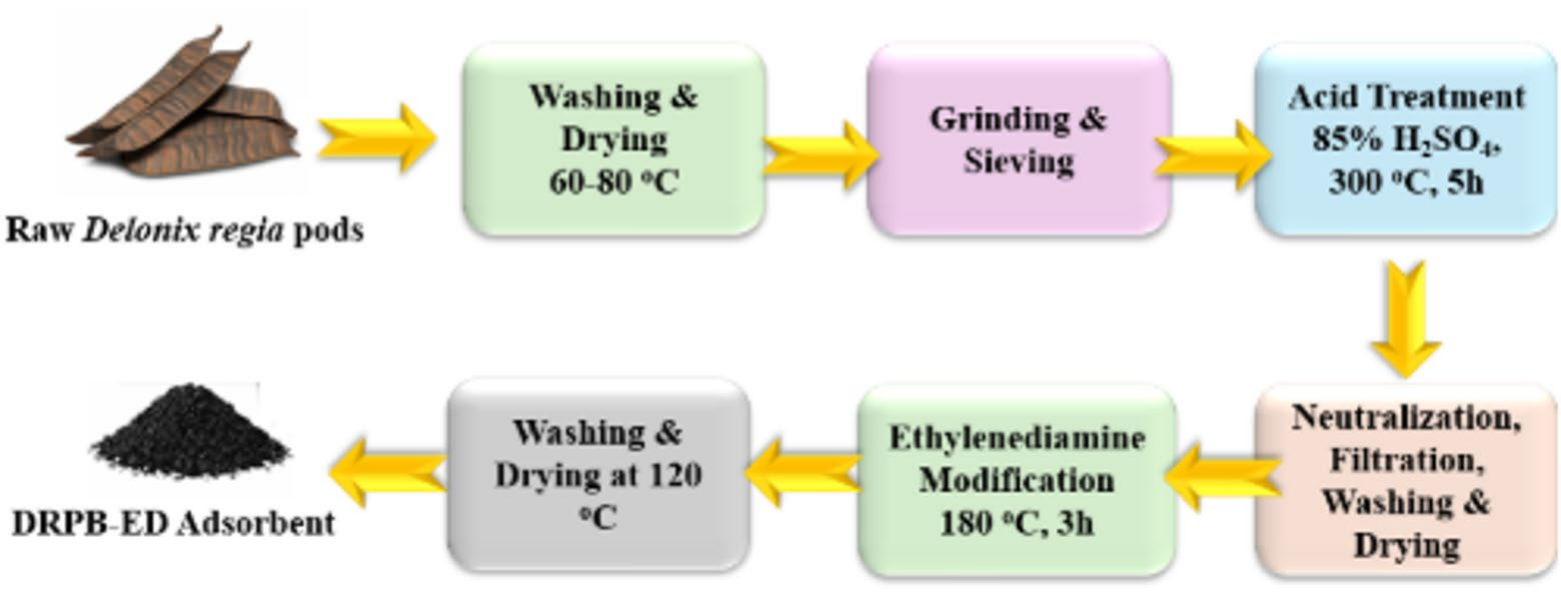
A schematic representation of the DRPB-ED synthesis route.

Raw material DRPs were used as the carbonaceous precursor for biochar synthesis. Prior to grinding and pulverization, the DRPs were repeatedly rinsed with tap water to remove dust and then dried in a furnace at 120 °C for 24 h. Powdered DRPs (100 g) were subjected to acid treatment in 500 mL of 85% H_2_SO_4_ at 300 °C for 5 h, after which the product was diluted, filtered, and repeatedly rinsed with distilled water until the pH approached neutrality^1^. After being washed with EtOH, the resulting biochar, known as DRPB-S (55 g), was dried in a furnace at 120 °C. Then, in a Teflon cup in a stainless autoclave container, 50 g of DRPB-S was combined with 150 mL of ethylenediamine, tightly sealed, and heated to 180 °C (hydrothermal treatment) for 3 h. To create DRPB-ED (58 g) as an adsorbent, the cooled mixture was diluted with distilled water, filtered, cleaned with distilled water and ethanol, and oven-dried at 120 °C for 24 h. A schematic representation of the DRPB-ED synthesis route is presented in Fig. 1.

### Batch adsorption

**Fig. 2:**
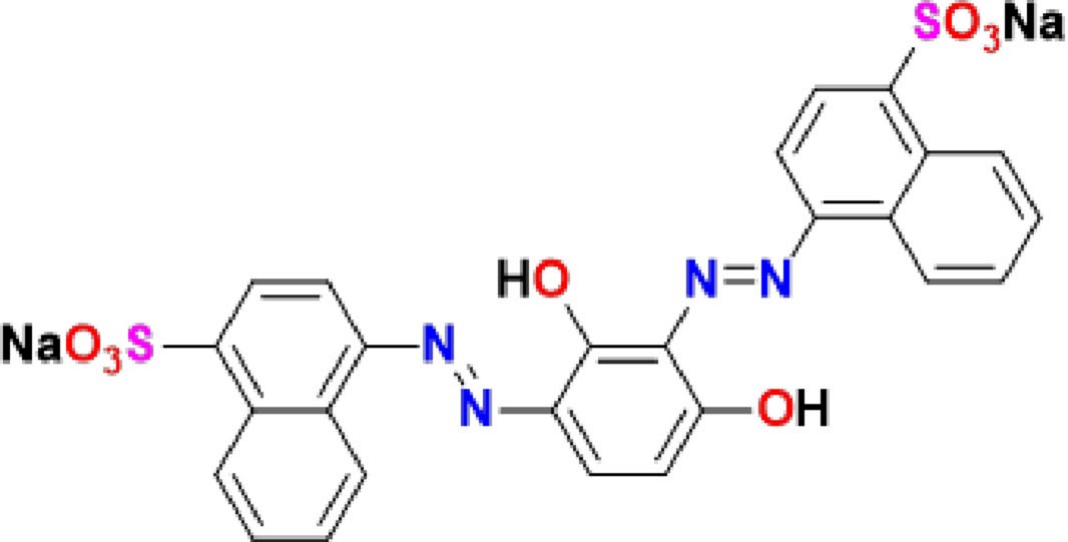
Molecular structure of the AB14 dye.

Batch adsorption studies were performed to investigate the adsorption efficacy and kinetics of DRPB-ED. A set of flasks was prepared, each containing 100 mL of AB14 dye solution at a different initial concentration (Fig. 2). DRPB-ED, added at different dosages, was stirred at 200 rpm for a specified duration. The pH of each solution was adjusted to predetermined levels by adding 0.1 M NaOH or HCl. Additionally, the solution pH was maintained at the target value throughout the adsorption equilibrium experiments. The concentration of AB14 dye was assessed by periodically extracting 0.1 mL samples from the solution and measuring them with a spectrophotometer calibrated at *λ*_max_ = 485 nm. DRPB-ED’s *q*_t_ was determined by applying Eq. (1).

1 $$\:{q}_{t}=\frac{\left({C}_{o}-{C}_{t}\right)V}{W}$$

Here, *C*_0_ (mg/L) signifies the initial concentration of AB14 dye; *C*_*t*_ (mg/L) denotes the dye concentration at time *t*; *q*_*t*_ (mg/g) denotes the adsorption capacity of DRPB-ED at time *t*; *W* (g) signifies the mass of the DRPB-ED, and *V* (L) represents the solution volume.

The effect of solution pH on AB14 adsorption was investigated by contacting 0.175 g of DRPB-ED with 100 mL of AB14 dye solution (100 mg/L) under continuous stirring at 200 rpm for 150 min at ambient conditions. Solutions with different initial AB14 dye concentrations (50–150 mg/L) were also prepared to evaluate adsorption isotherms and to assess the effect of DRPB-ED dosage on dye removal. AB14 dye concentrations were measured using AB14 dye solutions with different initial concentrations and DRPB-ED dosages ranging from 0.75 to 1.75 g/L. All samples were stirred at 200 rpm and 25 °C. The adsorption experiments were conducted in triplicate, with standard deviations ≤ ± 1.2; therefore, the data are expressed as mean values.

No background electrolyte was added during adsorption experiments; thus, ionic strength was not independently controlled and reflects only contributions from pH-adjusting agents and dissolved dye.

### RSM

Response surface methodology (RSM) was utilized to optimize the elements influencing AB 14 dye elimination in the presence of DRPB-ED. A D-optimal Design (DOD) was utilized, with Design-Expert version 13.0.5.0 as the software tool. The initial concentration of AB 14, the amount of adsorbent, and the reaction time were selected.

Table 1 enumerates the parameters under investigation alongside their respective levels. The proportion of AB 14 dye elimination (%) was the subject of inquiry. Seventeen experiments were conducted utilizing diverse combinations of variables.

**Table 1:** .

Factor	Name	Units	Minimum	Maximum	Coded low	Coded high	Mean	Std. Dev.
A	DRPB-ED	g/L	0.75	1.75	-1 ↔ 0.75	+ 1 ↔ 1.75	1.25	0.40
B	AD14 Dye	mg/L	50.0	150.0	-1 ↔ 50.0	+ 1 ↔ 150.0	103.13	42.55
C	Time	min	30.0	150.0	-1 ↔ 30.0	+ 1 ↔ 150.0	90.00	50.82

### ANN modeling

Artificial Neural Network (ANN) modeling predicts correlations between input and output variables by emulating the networks of the human brain. The feed-forward back-propagation neural network (BPNN), a widely used ANN architecture, is composed of an input layer corresponding to the independent variables, one or more hidden layers (HNs), and an output layer (OL) representing the dependent variable. The elimination of AB14 dye by the DRPB-ED ANN model is depicted using MATLAB R2015b, employing the Levenberg-Marquardt (LM) training algorithm, with training (70%), validation (15%), and testing (15%) datasets. The BPNN with a hidden layer of 4 neurons emerged as the optimal ANN model, with superior *R*^2^ and minimal MSE. The independent variables comprised the DRPB-ED adsorbent dosage (g/L), contact time (min), and initial AB14 dye concentration (mg/L), while AB14 dye removal efficiency was considered the dependent variable^35,36^.

RSM is a powerful statistical tool for experimental design, factor interaction analysis, and process optimization, providing a clear mathematical relationship between independent variables and responses, while simultaneously identifying optimal operating conditions within the studied range. Since real-world experimental systems often exhibit complex, nonlinear behavior, ANNs were also used as predictive models due to their superior ability to learn nonlinear patterns and improve prediction accuracy. Therefore, integrating RSM and ANNs offers a more comprehensive framework, with RSM contributing to interpretability and optimization, while ANNs enhance predictive performance. Furthermore, the combined use of both methods enables comparison and validation of results, thereby increasing the robustness and reliability of the modeling and optimization outcomes.

## Results and discussion

### DRPs, DRPB-S, and DRPB-ED characterization

**Fig. 3:**
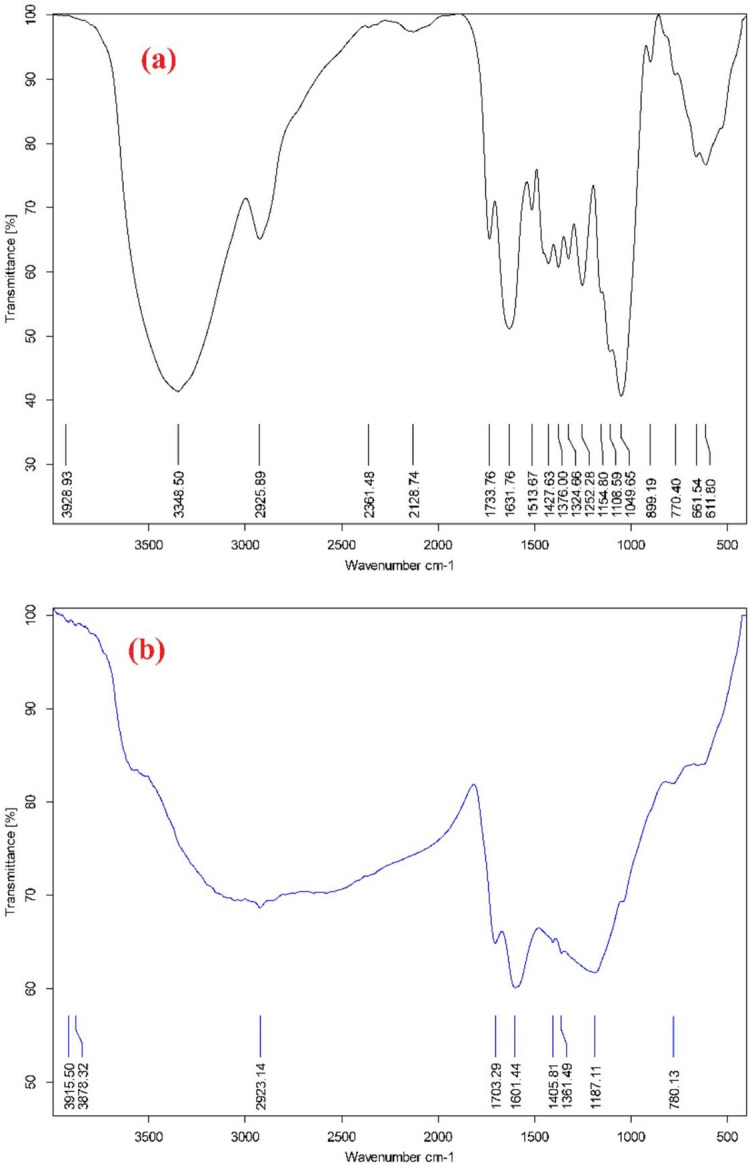

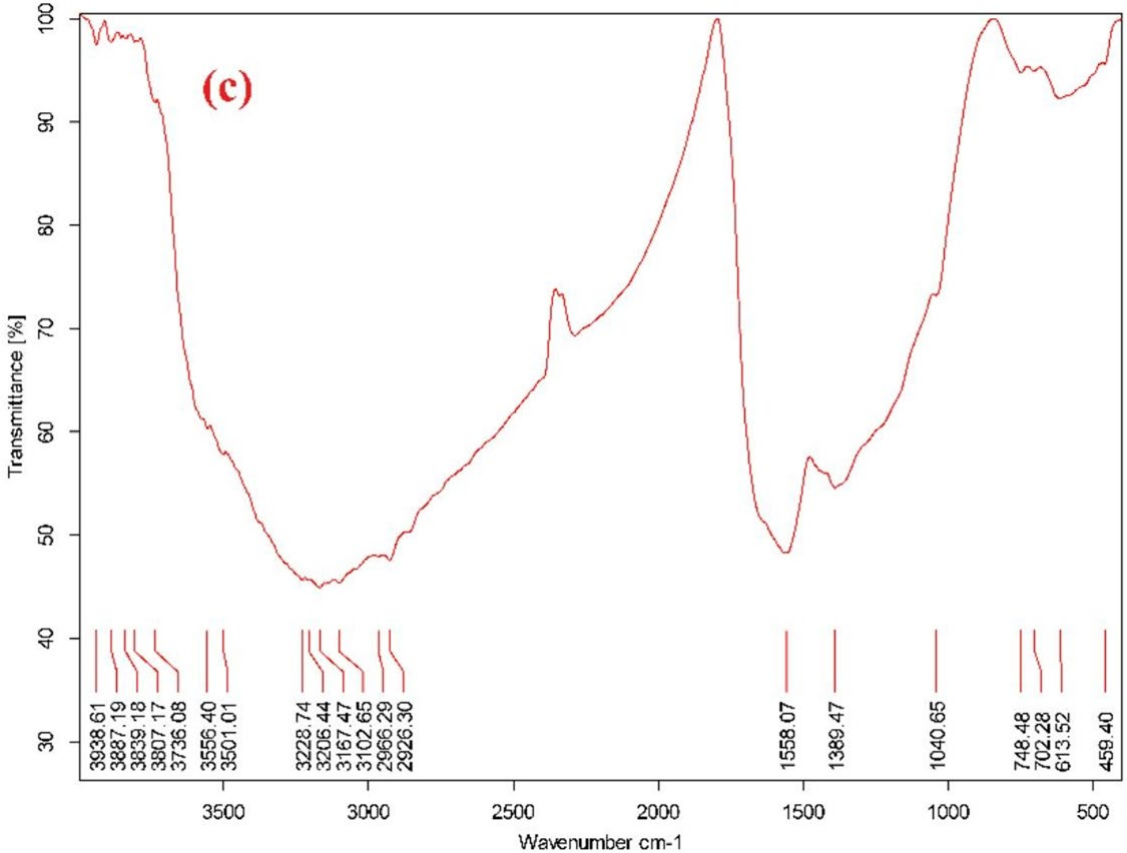
FTIR spectra of Delonix regia samples: (**a**) raw material (DRPs), (**b**) biochar (DRPB-S), and (**c**) ethylene diamine–functionalized hydrothermal biochar (DRBP-ED).

The surface functional groups present on DRPB-ED were identified through Fourier Transform Infrared (FT-IR) spectroscopy. Figures (3a and b) illustrates a comparative analysis of the FTIR spectra of DRPB-S and the raw DRPs. Differences in functional group composition among the materials were evident in the FT-IR spectra. In both DRPs and DRPB-S, the band observed between 3583.25 and 3348.50 cm^–1^ is attributed to the O–H stretching vibration (Fig. 3a,b). The prominent absorption peaks around 2925.89 cm^–1^ in DRPs (Fig. 3a) indicate the existence of –CH_2_ stretching groups, which shifted slightly to 2920.09 cm^–1^ in the expanded DRPB-S sample (Fig. 3b). The strong absorption band observed at 1733.76 cm^–1^ (Fig. 3a) corresponds to the C=O stretching vibration of ester functionalities in DRPs. For DRPB-S, this absorption band shifted to 1704.71 cm^–1^, indicating the formation of carboxyl functional groups (Fig. 3b). Compared with raw DPSPs, DRPB-S exhibits a stronger absorption at 1704.71 cm^–1^, indicating an enrichment of carbonyl (C=O) groups following sulfuric acid treatment^1,7,11^.

A characteristic band at 1631.76 cm^–1^ in the DRPs’ spectrum corresponded to the C=O stretching vibration of *β*-ketone groups. The observed shift to 1603.56 cm^–1^ with increased intensity in DRPB-S may correspond to the –C=C– stretching vibration (Fig. 3b). The peaks observed at 1513.67–1252.28 cm^–1^ in the DRPs’ spectrum correspond to the C–O functional group. In DRPB–S, the bands appearing at 1399.72 and 1367.29 cm^–1^, attributed to the S=O stretching vibration of sulfonyl groups, occupied the position of this band (Fig. 3b). Furthermore, the dehydration effect of H_2_SO_4_ facilitated the emergence of bands at 1182.88 and 1039.13 cm^–1^, which are associated with the formation of –SO_3_H and S=O functional groups in DRPB-S. These absorptions confirm that treating DRPs with H_2_SO_4_ resulted in the successful formation of DRPB-S. In addition, the DRPs exhibited a more prominent –C–O–C– asymmetric stretching vibration at 1049.65 cm^–1^ (Fig. 3a), whereas this band appeared with relatively lower intensity in DRPB-S^37–40^. The appearance and intensification of bands corresponding to –NH₂ stretching and bending vibrations confirm successful ethylenediamine functionalization. Under acidic conditions, these amine groups are readily protonated (–NH_2_ → –NH_3_^+^), generating positively charged surface sites. This protonation behavior is directly linked to the enhanced adsorption performance observed at lower pH values, indicating that electrostatic interactions contribute significantly to dye uptake.

Similar behavior has been reported for amine- and polymer-modified carbon materials, where nitrogen-containing functional groups significantly enhance surface polarity and electrostatic interaction capacity. For instance, polymer-modified carbon fiber systems exhibit comparable surface chemistry evolution upon amine incorporation, leading to improved adsorption efficiency^19^. Likewise, green carbon fiber-based materials demonstrate that surface functionalization alters electronic distribution and adsorption affinity^41^. These findings support the role of nitrogen functionalization in modulating the surface chemistry of DRPB-ED.

**Fig. 4:**
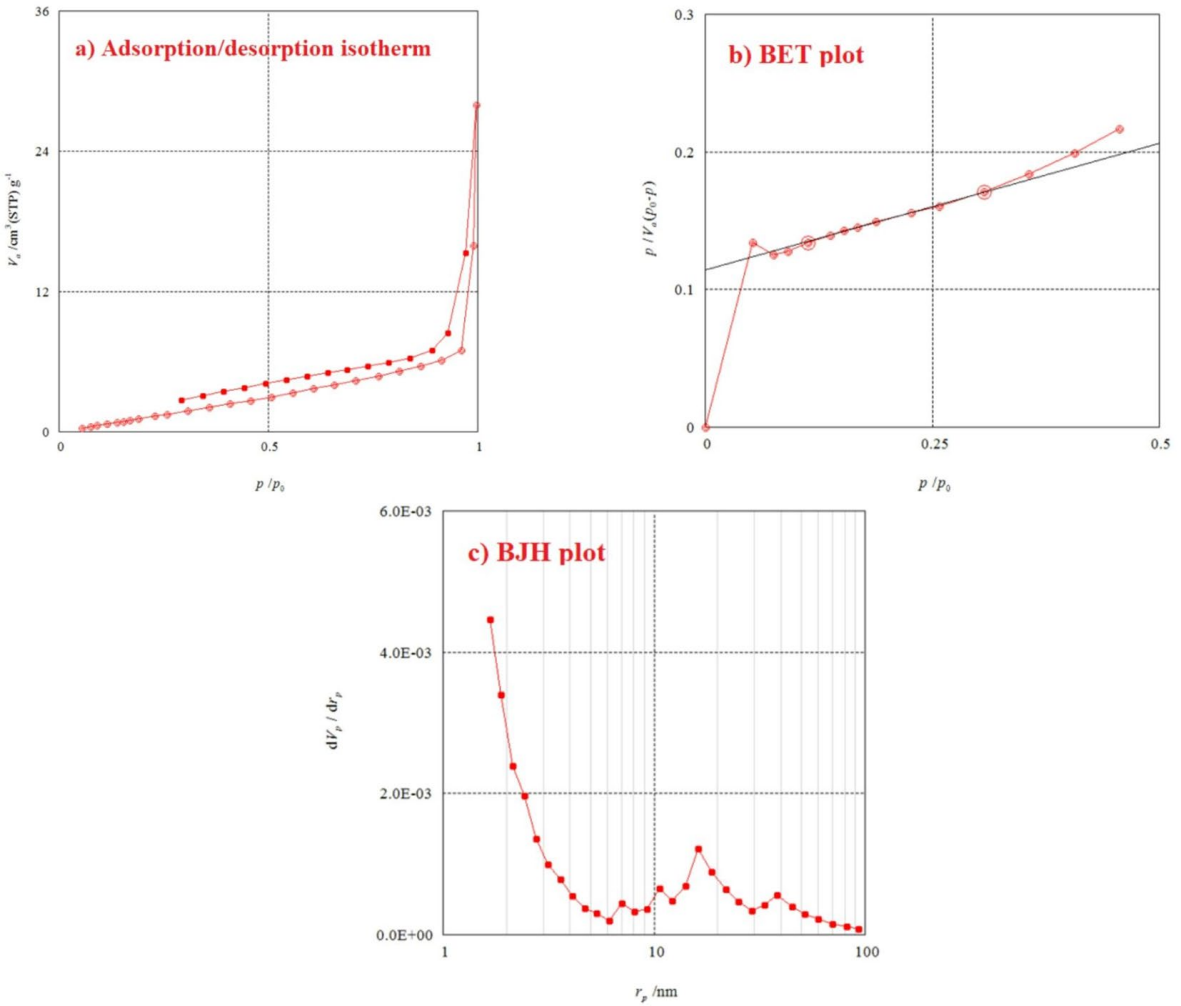
N_2_ adsorption–desorption isotherm (**a**), BET plot (**b**), and BJH pore size distribution (**c**) for DRPB-ED.

To investigate the impact of sulfuric acid modification on surface characteristics, the N₂ adsorption–desorption isotherm of DRPB-ED was analyzed. The specific surface area was assessed using the Brunauer–Emmett–Teller (BET) method, whereas mesoporous characteristics were evaluated using the Barrett–Joyner–Halenda (BJH) method. The resulting textural properties—BET surface area, mesopore area, total pore volume, average pore diameter, mesopore volume, mesopore distribution peak, and monolayer capacity—are presented in Fig. 4. A low specific surface area (14.745 m^2^/g) was observed for DRPB-ED based on BET analysis. The corresponding monolayer adsorption volume was calculated as 3.3878 cm^3^ (STP) g^–1^, while the total pore volume reached 1.8975 × 10^–2^ cm^3^/g. The average pore diameter of DRPB-ED was estimated to be 5.1474 nm. Moreover, the mesopore surface area, mesopore distribution peak, and mesopore volume were determined as 14.923 m^2^/g, 1.22 nm, and 2.1973 × 10^–2^ cm^3^/g, respectively^12,18^.

**Fig. 5:**
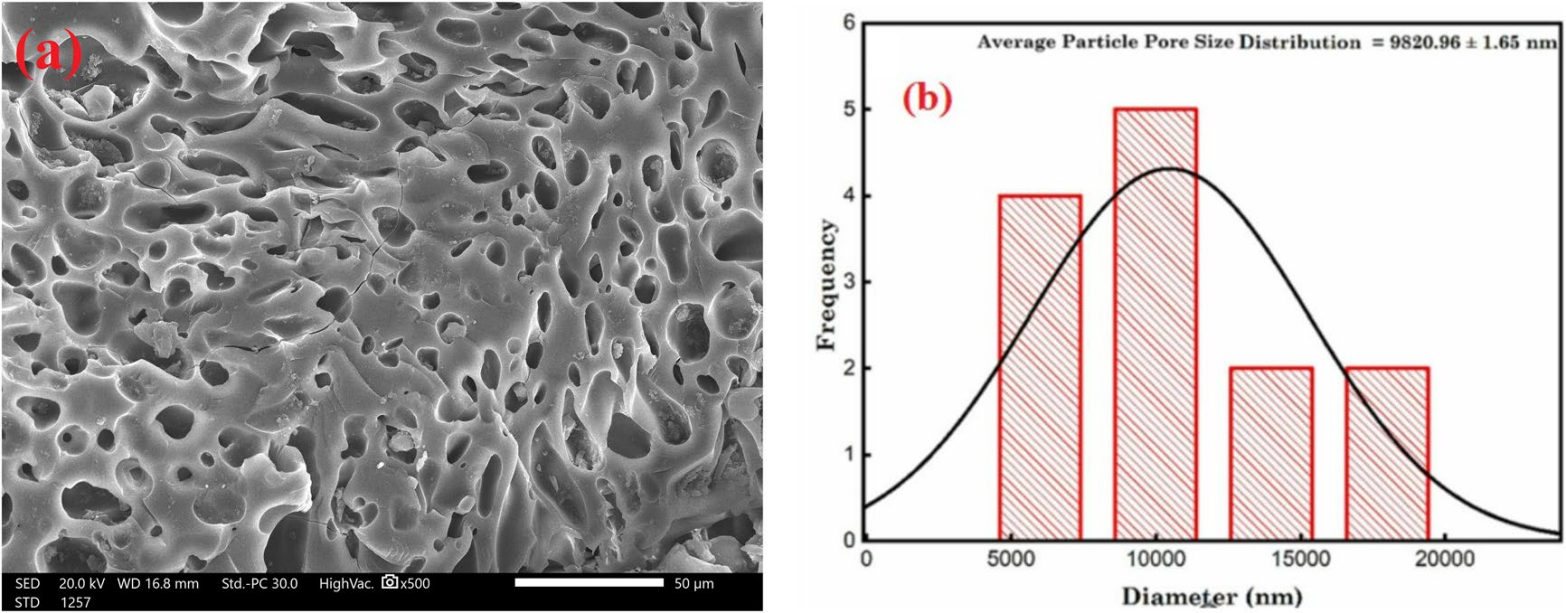
(**a**) High-vacuum SEM micrograph of DRPB-ED captured at ×700 magnification and 15.0 kV accelerating voltage, and (**b**) ImageJ analysis particle pore size distribution of DRPB-ED.

SEM micrographs reveal irregular surface cavities and large channel-like structures. Based on visual observation, these features are primarily attributed to macroporous structures (> 50 nm), as SEM resolution does not allow definitive identification of mesopores (2–50 nm). Mesoporosity is typically confirmed through nitrogen adsorption–desorption (BET) analysis rather than surface imaging alone. DRPB-ED’s SEM micrograph (Fig. 5a) reveals a rough, extremely heterogeneous surface with many interconnecting pores, fissures, and cavities that range in dimension from submicrometers to several micrometers. The vast accessible surface area and numerous active sites provided by this intricate three-dimensional network of macropores and channels are expected to promote the penetration and diffusion of AB14 dye molecules into the particles’ cores and to increase overall adsorption capacity. ImageJ analysis revealed that DRPB-ED exhibited an average pore size of 9821 ± 1.65 nm, with pore diameters ranging from 4072 to 17,200 nm, indicating a broad particle pore size distribution (Fig. 5b). This suggests that the majority of pores fall into the meso-to macroporous range and so aid in quick mass transfer and effective dye removal^11,12,18^.

Although the BET surface area of DRPB-ED is relatively moderate, the high adsorption capacity observed cannot be attributed solely to textural properties. In functionalized carbon materials, adsorption performance is often governed more by surface chemistry than by surface area alone. The presence of abundant amine groups on DRPB-ED enhances electrostatic attraction, hydrogen bonding, and possible donor–acceptor interactions with azo dye molecules. Therefore, the adsorption process appears to be dominated by specific chemical interactions rather than by purely physical surface-area effects. Similar observations have been reported in nitrogen-functionalized carbon adsorbents, where enhanced adsorption capacity was achieved despite moderate BET surface areas.

It should be noted that pore-size observations from SEM images are qualitative and primarily reflect surface morphology, whereas BJH analysis provides a quantitative pore-size distribution derived from nitrogen adsorption–desorption isotherms. SEM typically visualizes larger surface openings (macropores), whereas BJH analysis is sensitive to mesopores in the 2–50 nm range. Therefore, slight discrepancies between SEM-based visual estimations and BJH pore size distributions are expected and do not indicate inconsistency but rather reflect the different measurement principles and pore-size sensitivities of the two techniques^11,12^.

The pore features observed in the SEM images should be interpreted as qualitative morphological evidence rather than as a rigorous quantitative measure of pore size distribution. SEM primarily reveals surface texture and larger visible pore openings, which are generally associated with surface-accessible macropores or larger structural voids. In contrast, the BJH method provides a quantitative pore-size distribution derived from nitrogen adsorption–desorption isotherms and is particularly appropriate for analyzing mesopores in the 2–50 nm range. Accordingly, slight differences between SEM-based visual observations and BJH-derived pore-size values are expected, as the two techniques rely on distinct characterization principles and are sensitive to distinct pore-size domains. Thus, SEM and BJH should be regarded as complementary rather than contradictory tools for characterizing porous structures.

### Effect of pH

**Fig. 6:**
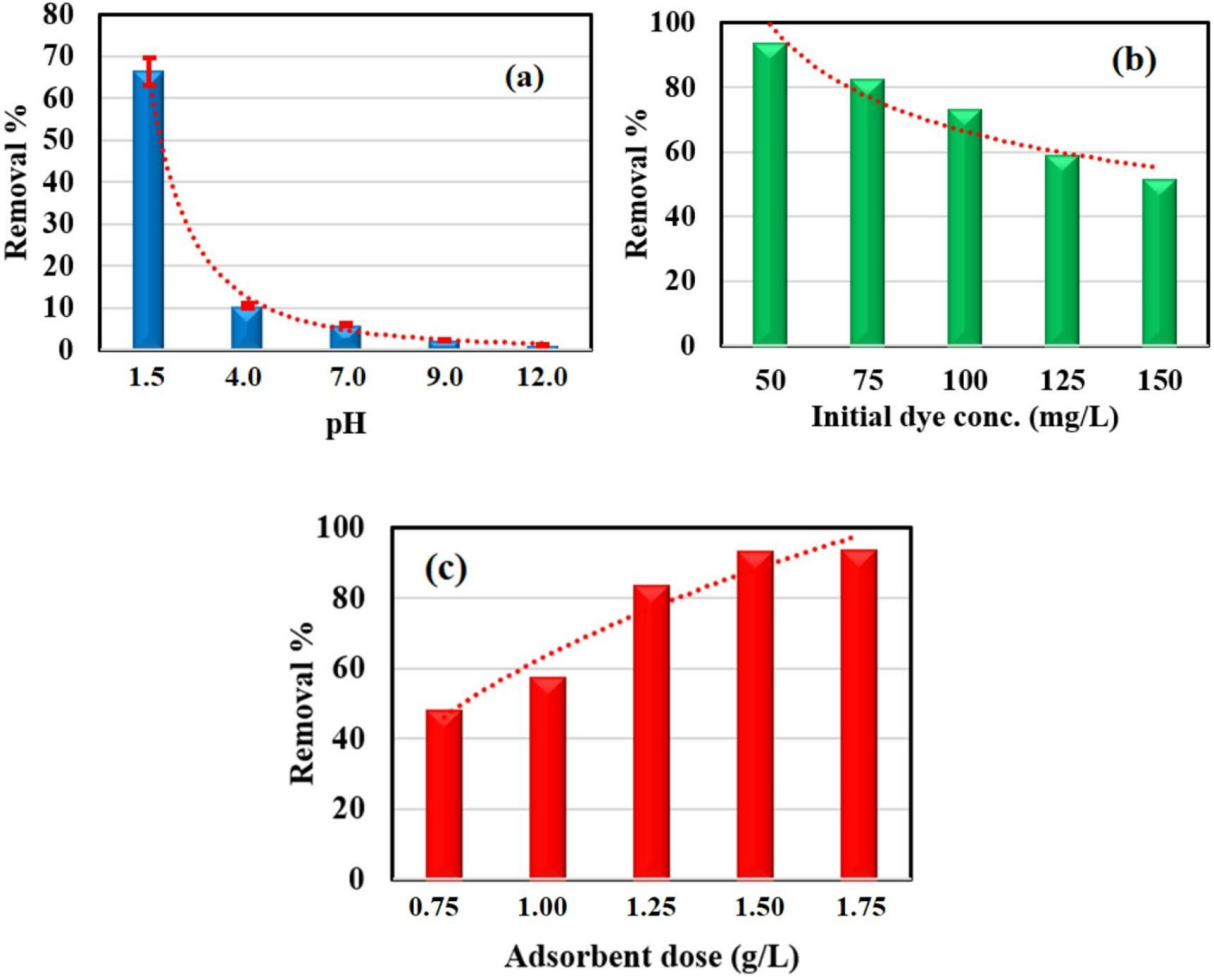
Effect of (**a**) pH (adsorbent dosage: 1.75 g/L; initial AB14 concentration: 100 mg/L; contact time: 150 min; temperature: 25 °C), (**b**) initial dye concentration (adsorbent dosage: 1.75 g/L; pH: 1.5; contact time: 150 min; temperature: 25 °C), and (**c**) adsorbent dosage (initial AB14 concentration: 150 mg/L; pH: 1.5; contact time: 150 min; temperature: 25 °C) on AB14 dye removal by DRPB-ED.

The adsorption of AB14 dye onto DRPB-ED was strongly influenced by the solution pH (Fig. 6a). Maximum adsorption efficiency (66.5%) was observed at a pH of 1.5, where the acidic environment promoted the protonation of functional groups on the DRPB-ED surface, rendering it positively charged. This positive surface charge facilitated strong electrostatic interactions with the negatively charged AB14 dye molecules, enhancing adsorption efficiency. As the pH increased, a significant decline in adsorption efficiency was observed. At pH 7, the removal efficiency dropped to 6.0%, and at pH 12, it further decreased to 1.3%. This reduction is attributed to the deprotonation of DRPB-ED’s surface functional groups at higher pH, resulting in a negatively charged surface. The resulting electrostatic repulsion between the negatively charged surface and AB14 dye molecules diminishes the adsorption capacity. This pH-dependent trend underscores the critical role of solution pH in optimizing adsorption processes. Under acidic conditions, the surface of DRPB-ED not only enhances electrostatic attraction but also potentially facilitates hydrogen bonding interactions with AB14 molecules. Conversely, the decreased efficiency at neutral and alkaline pH levels highlights the importance of controlling pH for effective industrial dye removal. Similar studies on modified biochar adsorbents have reported maximum adsorption capacities at acidic pH, typically 2.0. For instance, mandarin peel biochar achieved peak adsorption efficiency for AB14 at low pH, further emphasizing the importance of surface protonation^42^. Compared to other biochar-based adsorbents, DRPB-ED demonstrates performance comparable to or superior to theirs, positioning it as a viable option for pH-sensitive wastewater treatment applications.

Although the point of zero charge was not experimentally determined, the surface charge behavior can be inferred from pH-dependent adsorption results. The enhanced adsorption under acidic conditions suggests protonation of amine functionalities (–NH_2_ → –NH_3_^+^), generating positively charged active sites that favor electrostatic attraction with anionic dye species. This confirms that electrostatic interactions play a dominant role in the adsorption mechanism. The introduction of nitrogen-containing functional groups via ethylenediamine modification significantly alters the surface charge distribution of DRPB-ED, thereby enhancing its electrostatic interaction capability relative to pristine DRPB-S.

Although maximum adsorption was observed at pH 1.5, the practical feasibility of operating at highly acidic conditions should be carefully considered. Industrial wastewater treatment systems rarely operate at such low pH values due to corrosion risks, chemical consumption, and post-treatment neutralization requirements. Therefore, while acidic conditions enhance electrostatic attraction via protonation of amine groups, practical implementation would require evaluation of cost–benefit balance and system durability. In practical scenarios, partial pH adjustment or integration with existing acidic effluent streams may improve feasibility. Alternatively, surface modification strategies aiming to broaden the effective pH window could enhance industrial applicability.

Several biomass-derived activated carbons have demonstrated efficient dye removal over a pH range from moderately acidic to neutral. For example, biomass-derived activated carbon systems optimized via response surface methodology have shown significant adsorption performance at higher pH values^17^. Compared to such materials, DRPB-ED exhibits superior adsorption under strongly acidic conditions due to enhanced amine protonation, although its optimal performance window is narrower.

Although pH 1.5 yielded the highest adsorption efficiency, this condition primarily represents an ideal laboratory setting for optimizing adsorption performance and understanding the underlying mechanism. The protonation of the adsorbent surface, which promotes stronger contact with the adsorbate, is responsible for the increased removal under very acidic conditions. However, due to the expense and operational demands of acid addition and subsequent neutralization, such a low pH may impose practical restrictions in full-scale treatment. Therefore, rather than a direct prescription for industrial operations, the optimal pH identified in this work should be viewed as a mechanistic and performance benchmark. In real-world applications, the initial properties of the wastewater will determine its viability, especially for companies producing naturally acidic effluents. However, operating in less acidic settings may offer a more practical balance between economic viability and efficiency.

### Effect of initial dye concentration

The enhanced adsorption characteristics of AB14 dye onto DRPB-ED were examined at varying initial dye concentrations to determine optimal adsorption conditions. The results demonstrated a clear dependency, with significant implications for industrial applications. Increasing the initial concentration of AB14 dye led to a decrease in percentage removal (%), as shown in Fig. 6b. Although higher initial concentrations enhance the mass transfer driving force and may increase the absolute adsorption capacity (*q*_*e*_, mg/g), the overall percentage removal declines due to the finite number of available active sites on the DRPB-ED surface. At elevated dye concentrations, these adsorption sites progressively approach saturation, limiting the fraction of dye that can be removed relative to the total amount present in solution. Thus, while the amount of dye adsorbed per unit mass of adsorbent may increase, the removal efficiency (%) decreases due to site limitation. This distinction highlights the importance of differentiating between adsorption capacity and percentage removal when evaluating adsorption performance.

### Effect of adsorbent dosage

The impact of adsorbent dosage was assessed between 0.75 and 1.75 g/L, revealing a proportionate increase in removal efficiency with dosage. Maximum removal efficiency of 93.23% was achieved at a dosage of 1.75 g/L, as shown in Fig. 6c. At higher dosages, the improvement in removal efficiency became less pronounced. This behavior can be attributed to several factors, including particle agglomeration at elevated solid concentrations, which may reduce the effective surface area available for adsorption. Additionally, at relatively low solute concentrations, a significant fraction of adsorption sites may remain unsaturated, limiting the proportional increase in removal efficiency. Equilibrium constraints and possible mass transfer limitations within the batch system may also contribute to this trend. These findings highlight the importance of optimizing adsorbent dosage to achieve maximum removal efficiency while ensuring efficient utilization of adsorption sites. These results emphasize the need to carefully optimize both the initial dye concentration and the adsorbent dosage to achieve maximum adsorption efficiency while minimizing material waste. DRPB-ED’s high efficiency at moderate dosages and concentrations makes it a promising candidate for cost-effective and scalable wastewater treatment applications.

### Adsorption Isotherms

Adsorption isotherm models were employed to evaluate the adsorption capacity and mechanism of AB14 dye on DRPB-ED. The experimental data were evaluated using the Langmuir (LIM), the Freundlich (FIM), and the Temkin (TIM) models to provide a comprehensive characterization of the adsorption behavior (Table 2). The LIM, which assumes monolayer adsorption on uniform surfaces, yielded the best fit to the experimental data. The maximum adsorption capacity (*Q*_m_) of DRPB-ED for AB14 was approximately 60 mg/g, indicating a strong affinity between the adsorbent and the dye. The Langmuir constant (*K*_*L*_), as presented in Table 2, represents the intensity of the interaction between the adsorbate and the adsorbent. These results highlight the efficiency of DRPB-ED in removing AB14 dye and its potential application in wastewater treatment systems. The Freundlich model, describing adsorption on heterogeneous surfaces, provided additional insights. The Freundlich constants (*K*_F_) and intensity parameters (*n*), also listed in Table 2, suggest that DRPB-ED exhibits surface heterogeneity due to functional groups introduced during ethylenediamine modification. This heterogeneity broadens the range of concentrations over which DRPB-ED is effective, making it versatile for practical applications. The intensity parameter (*n* > 1) further indicates favorable adsorption conditions. The Temkin model, which accounts for indirect adsorbate-adsorbate interactions, showed that the heat of adsorption declines linearly with increasing surface coverage. The parameters *A*_*T*_ and *B*_*T*_, shown in Table 2, suggest that the adsorption process is energetically favorable and supports efficient dye removal across a range of equilibrium concentrations. To evaluate the accuracy of each model, error functions such as hybrid error function, average percentage error (APE%), chi-square test (X²), absolute error (EABS), error sum of squares (ERRSQ), mean percentage square deviation (MPSD), and root mean square (RMS) were calculated. Among these, the Langmuir model consistently yielded the lowest error values, reinforcing its suitability for describing adsorption behavior. These findings confirm that adsorption occurs via a monolayer mechanism with high efficiency on the surface of DRPB-ED.

**Table 2:** Adsorption isotherm data for AB14 dye on DRPB-ED at 25 °C with varying dye concentrations (50–150 mg/L) and adsorbent doses (0.75–1.75 g/L).

Isotherm model	Parameters	DRPB-ED doses (g/L)				
		0.75	1.00	1.25	1.50	1.75
LIM	*Q*_*m*_ (mg/g)	60.61	58.82	47.17	46.51	45.25
	*K*_*L*_ x 10^3^	0.038	0.042	0.278	0.499	0.334
	*R* ^*2*^	0.996	1.000	0.999	0.999	0.999
FIM	*Q*_*m*_ (mg/g)	44.0	44.2	47.3	46.8	47.0
	*K*_*F*_	11.7	10.3	25.9	27.5	21.9
	*R* ^*2*^	0.999	0.977	0.915	0.907	0.954
TIM	*A*_*T*_	0.492	0.447	94.414	274.183	37.541
	*B*_*T*_	12.297	12.638	5.122	4.674	5.643
	*b*_*T*_	201.478	196.041	483.759	530.053	439.060
	*R* ^*2*^	0.995	0.994	0.920	0.919	0.959

**Fig. 7:**
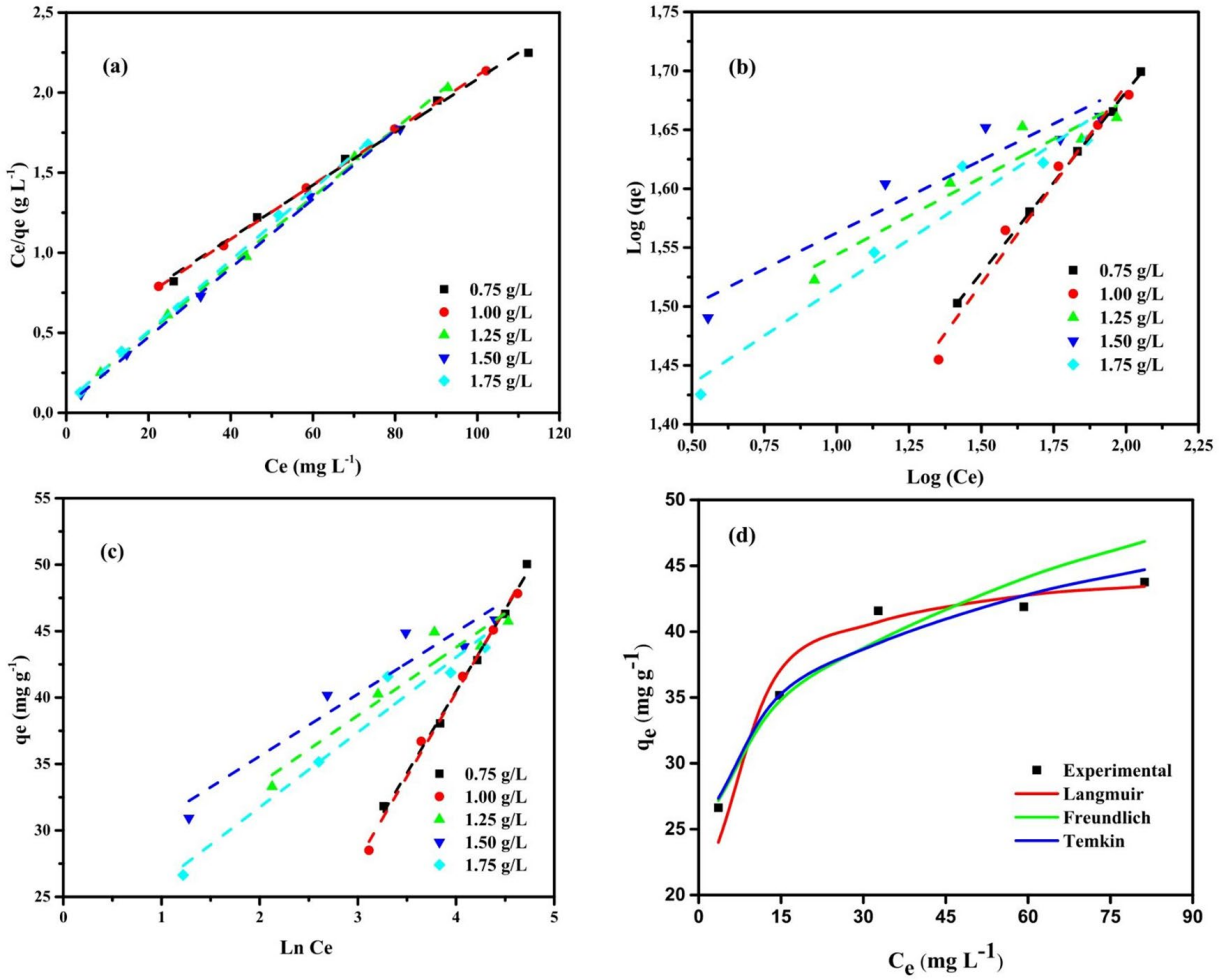
LIM (**a**), FIM (**b**), TIM (**c**), and comparison of observed and predicted isotherms (**d**) for AB14 dye on DRPB-ED at 25 °C with dye concentrations of 50–150 mg/L and adsorbent doses of 0.75–1.75 g/L.

The Langmuir model closely matches the experimental data, as shown in the comparative plot of the isotherm models in Fig. 7a. The decrease in *Q*_m_ at higher adsorbent dosages is attributed to adsorbate-limited conditions arising from the relatively low initial dye concentration, rather than to a reduction in the adsorbent’s intrinsic adsorption capacity. Even though the Freundlich and Temkin models differ somewhat at higher equilibrium concentrations, they nevertheless offer insightful supplementary information about the adsorption behavior of DRPB-ED (Fig. 7). Furthermore, DRPB-ED’s performance was contrasted with that of other adsorbents based on biochar. The enhanced adsorption performance of DRPB-ED is demonstrated by its higher *Q*_m_ of 60.61 mg/g, which is likely due to improved surface characteristics and increased functional group availability resulting from ethylenediamine modification.

For comparison, Elbager et al.^17^ reported significantly higher maximum adsorption capacities for different adsorbent systems. In their study on activated carbon derived from Desert Palm Kernel Shells (DDSSAC), a *Q*_m_ value of 716 mg/g was achieved for methylene blue removal, which was attributed to multiple interaction mechanisms, including hydrogen bonding, π–π stacking, hydrophobic interactions, and electrostatic attraction^17^. In another work, Elbager et al.^19^ synthesized a copolymer-modified composite (PMMGCF) from Arabian palm leaves, obtaining a maximum adsorption capacity of 411.71 mg/g for Congo Red dye^19^, demonstrating the positive effect of advanced polymer functionalization strategies.

Although the reported adsorption capacities in these studies are higher, it is important to note that adsorption performance is strongly influenced by factors such as the nature of the target pollutant (e.g., molecular size, charge, and structure), surface chemistry, pore structure, synthesis route, and modification strategy. Unlike the extensively polymer-modified or highly activated systems reported by Elbager et al.^19^, DRPB-ED was prepared via a comparatively straightforward ethylenediamine functionalization approach, offering a balanced combination of adsorption efficiency, material simplicity, and potential cost-effectiveness. Therefore, the obtained *Q*_m_ value of 60.61 mg/g demonstrates that DRPB-ED is a competitive and promising adsorbent among amine-functionalized carbon materials, particularly with respect to synthesis feasibility and surface engineering strategy.

### Analysis of statistical error functions in isothermal models

Following the acquisition of empirical equilibrium data for AB14 dye on DRPB-ED through LIM, FIM, and TIM, various error functions were employed, including average percent errors (APE), chi-square error (*X*^2^), hybrid error function (HYBRID), Mar quardt’s percent standard deviation (MPSD), the sum of the absolute errors (EABS), and the root mean square errors (RMS). As shown in Table 3, smaller error function values demonstrate closer correspondence between the experimental data for DRPB-ED and the isotherm model predictions. Although the most suitable model according to *R*^2^ values was LIM, the TIM model showed excellent agreement with the experimental results, as indicated by the error function values in Table 3.

Although the LIM model exhibited the highest *R*^2^ value, indicating a strong linear correlation, the error function analysis suggested slightly better agreement for the TIM model. This apparent discrepancy arises because *R*^2^ alone assesses the goodness of fit, whereas error functions provide a more sensitive measure of the deviation between experimental and calculated values. Therefore, model selection should not rely solely on *R*^2^ but also consider statistical error parameters and the physical assumptions of each model.

In the present study, although the Temkin model yielded lower error values, the Langmuir model was considered more appropriate due to its theoretical assumption of monolayer adsorption on a homogeneous surface, which is consistent with the observed saturation behavior and a chemisorption-dominated mechanism. Hence, the Langmuir model was selected as the most representative isotherm, while the Temkin model also provides complementary insight into adsorbate–adsorbent interactions.

**Table 3:** Error function values for isotherm models applied to AB14 dye adsorption on DRPB-ED.

Model	APE%	X^2^	Hybrid	ERRSQ	MPSD	EABS	RMS
LIM	0.009	0.006	0.024	0.224	0.049	2.367	0.047
FIM	0.032	0.061	0.266	2.366	0.166	7.691	0.159
TIM	0.000	0.000	0.000	0.000	0.000	0.001	0.000

### Adsorption kinetics

**Fig. 8:**
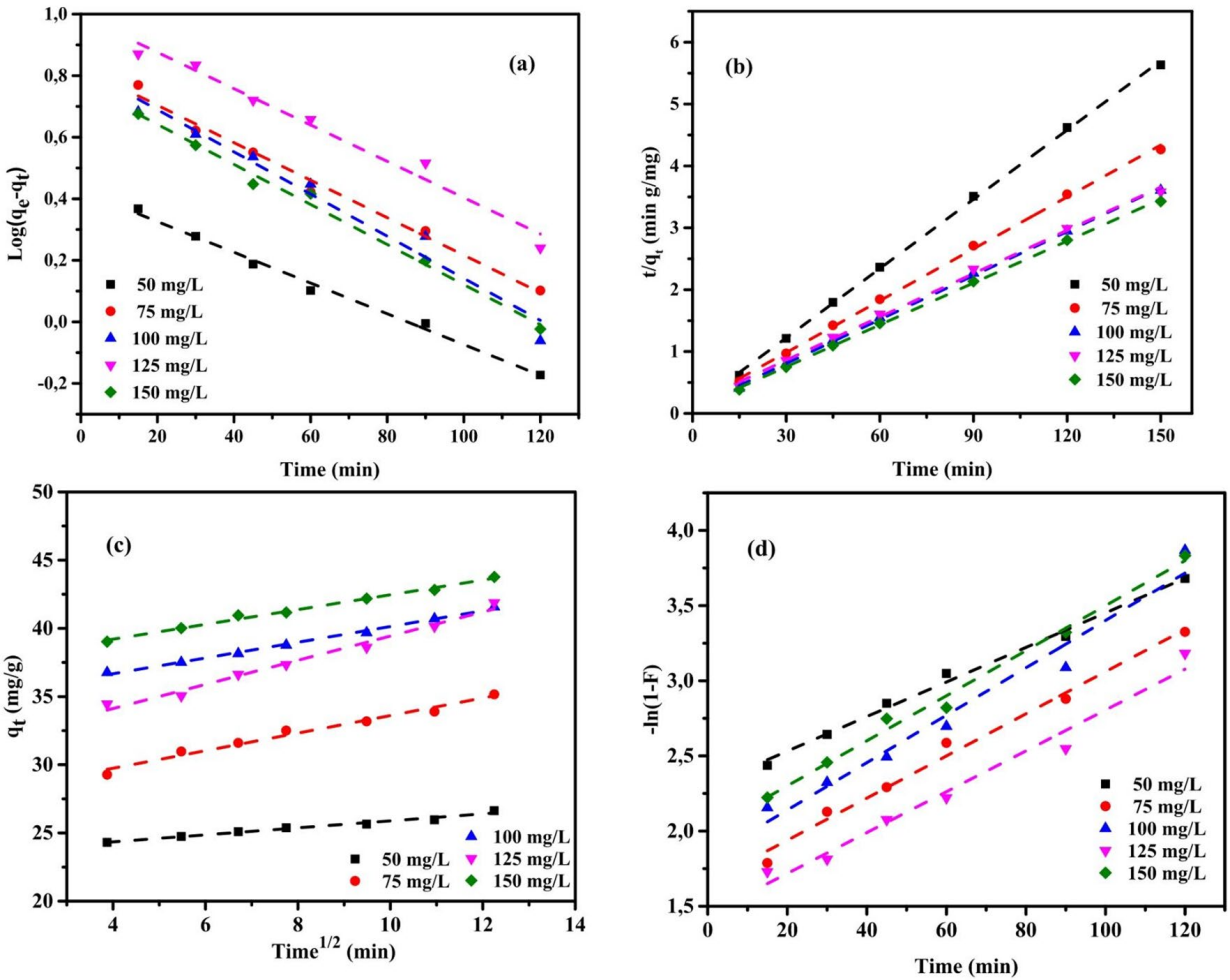
The plot of (**a**) PFOM, (**b**) PSOM, (**c**) IPDM, and (**d**) FDM of adsorption of AB14 dye by DRPB-ED adsorbent (*C*_0,_ AB14 dye = (50–150 mg/L), *C*_0_, DRPB-ED = (1.75 g/L), Temp. = 25 °C).

An in-depth kinetic investigation of AB14 dye adsorption onto DRPB-ED was conducted using multiple models encompassing diffusion, mass transfer, and chemical reaction mechanisms. This analysis aimed to determine the optimal operating parameters for a potential batch-scale application^43^. Understanding kinetic parameters in depth is fundamental to describing adsorption behavior and is key to the design and optimization of adsorption systems. To analyze the removal performance of AB14 dye by DRPB-ED, kinetic behavior was examined using the pseudo-first-order (PFOM)^44^, pseudo-second-order (PSOM)^45^, intraparticle diffusion (IPDM)^46^, and film diffusion (FDM)^47^ models. The agreement between experimental data and model predictions was evaluated using the correlation coefficient (*R*^2^), as illustrated in Fig. 8 and summarized in Tables 4 and 5.

#### Pseudo-first-order model (PFOM)

The adsorption rate constant was determined using the Lagergren pseudo-first-order model (PFOM)^44^, which provides a preliminary description of adsorption kinetics based on adsorption capacity. The corresponding kinetic equation is presented in Eq. (2).

2 $$\:\mathrm{log}\left({q}_{\mathrm{e}}-{q}_{\mathrm{t}}\right)=\mathrm{log}\left({q}_{\mathrm{e}}\right)-\frac{{k}_{1}}{2.303}t$$

The variables *q*_t_ and *q*_e_ (mg/g) represent the amounts of ions adsorbed at time *t* and at equilibrium, respectively. The parameter *k*_*1*_ (min^–1^) represents the adsorption rate constant associated with the PFOM. As illustrated in Fig. 8a, the parameters *k*_*1*_ and *q*_*e*_ were obtained from the slope and intercept of the linear plot of log (*q*_*e*_*-q*_*t*_) versus time. As illustrated in Table 4, the correlation coefficients (*R*^2^) for the kinetic models range from 0 to 1, with values close to 1 indicating stronger agreement between the models and the experimental data. The consistently high coefficients of determination (*R*^2^ > 0.893) indicate strong agreement between the experimental and predicted equilibrium adsorption capacities (*q*_e_), confirming that the PFOM accurately represents the adsorption behavior of AB14 dye onto DRPB-ED. Moreover, as shown in Table 4, no systematic increase or decrease in R² values was observed as the DRPB-ED dosage increased from 0.75 to 1.75 g/L.

#### Pseudo-second-order model (PSOM)

In this work, the adsorption performance of DRPB-ED in removing AB14 dye was evaluated using the pseudo-second-order kinetic model (PSOM). In the pseudo-second-order model, adsorption kinetics are governed by the square of the number of unoccupied surface active sites. Moreover, the amount of solute adsorbed influences the overall reaction kinetics. The corresponding kinetic expression for the PSOM is provided in Eq. (3).

3 $$\:\left(\frac{t}{{q}_{t}}\right)=\frac{1}{{k}_{2}{q}_{e}^{2}}+\frac{1}{{q}_{e}}\left(t\right)$$

where *k*_2_ (g mg^–1^ min^–1^) denotes the equilibrium rate constant for PSOM adsorption.

Application of the PSOM yields a linear relationship between *t/q*_*t*_ and *t*. As shown in Fig. 8b, the slope and intercept of the linear $\:t/{q}_{t}$ versus * t* plot were used to determine the rate constant ($\:{k}_{2}$) and equilibrium adsorption capacity ($\:{q}_{e}$), respectively, for AB14 adsorption onto DRPB-ED. The PSOM-derived parameters, including *k₂*, the theoretical and experimental *q*_e_ values, and the correlation coefficients (*R*^2^), are summarized in Table 4. The results show that the PSOM yields coefficients of determination (*R*^2^) close to unity, indicating excellent agreement between calculated and experimental adsorption capacities over the entire range of initial dye concentrations. The adsorption mechanism of organic dyes generally involves physical interactions such as electrostatic attraction, complex formation, hydrogen bonding, π–π stacking, pore filling, and network entanglement. Under acidic conditions, the adsorption of anionic dyes is predominantly driven by electrostatic attraction between protonated amino groups and dye anions. Conversely, in alkaline media, the reduced protonation of –NH_3_^+^ groups suppresses electrostatic attraction, leading to a mechanism dominated by chemical adsorption^48^. These observations confirm that the PSOM accurately represents the kinetic behavior of AB14 dye adsorption onto DRPB-ED, rendering it the most suitable model among those investigated.

The dominance of the PSOM kinetic model suggests that chemisorption governs adsorption. In the case of DRPB-ED, this behavior can be attributed to specific interactions between protonated amine groups (–NH_3_^+^) and azo dye molecules. Beyond electrostatic attraction, possible mechanisms include hydrogen bonding and electron donor–acceptor interactions between nitrogen-containing functional groups and azo (–N=N–) moieties. Similar PSOM-dominated adsorption behavior has been reported for nitrogen-functionalized carbon systems, where surface amine groups facilitate strong chemical interactions with dye molecules^41^. Likewise, chemisorption-driven dye-removal mechanisms have been documented in polymer-modified carbon adsorbents with abundant amine functionalities^19^. These findings support the interpretation that the adsorption process in DRPB-ED is not purely diffusion-controlled but involves surface chemical interactions.

#### Intra-particle diffusion model (IPDM)

Adsorption of pollutants onto solid adsorbents typically occurs through a sequence of four steps: (i) transport of solute species from the bulk solution to the external surface of the adsorbent by bulk diffusion; (ii) migration of solute molecules across the liquid film surrounding the adsorbent particles (film diffusion); (iii) diffusion of pollutants into the internal pores of the adsorbent (intraparticle diffusion), enabling interaction with internal surfaces; and (iv) fixation of pollutants at active sites via mechanisms such as complexation, ion exchange, or chelation^46^. According to Kargi and Çikla, the intraparticle diffusion model is a widely adopted approach for interpreting mass transfer mechanisms between liquid solutions and solid adsorbents^49^. In batch systems with strong agitation, the isothermal plug-flow diffusion mechanism can be rate-determining in the adsorption process^50^. The application of Eq. (4), as proposed by Annadurai et al.^51^, was used to further interpret the IPDM behavior under these conditions.

4 $$\:{q}_{t}={K}_{diff}{t}^{0.5}+C$$

where *K*_*diff*_ signifies the rate constant of IPDM (mg g^–1^ min^1/2^) (Fig. 8c).

The Weber–Morris model^52^ postulates that intraparticle diffusion governs the adsorption process when the plot of *q*_*e*_ versus *t*^1/2^ passes through the origin, as depicted in Fig. 8c. In contrast, when the plot does not intersect the origin and exhibits a pronounced intercept (*C*), the adsorption rate is typically governed by film diffusion. This study investigated the adsorption behavior of AB14 dye onto DRPB-ED under various operating conditions, including changes in adsorbent dosage and initial dye concentration. The intraparticle diffusion of AB14 dye was analyzed using Weber–Morris plots^52^(Fig. 8c), with the derived diffusion rate constants (*K*_*dif*_) and intercepts (*C*) calculated from the linear $\:{q}_{t}$–$\:{t}^{1/2}$ relationships and presented in Table 5. The fact that these lines do not pass through the origin indicates a significant intercept, implying that film diffusion contributes notably to the overall rate control of AB14 dye adsorption onto DRPB-ED. As shown in Fig. 8d, the adsorption rate increases gradually with time. The intraparticle diffusion rate constant (*K*_dif_) ranged from 0.247 to 3.058 mg g^–1^ min^–0.5^, with higher values observed at higher initial dye concentrations and lower adsorbent dosages. As adsorption proceeds, the diminishing surface area and pore volume of DRPB-ED explain the observed decrease in adsorption rate.

#### Film diffusion model (FDM)

The movement of adsorbate species through the boundary layer around adsorbent particles is represented by the film diffusion model (FDM)^47^. This mechanism is expressed mathematically in Eq. (5).

5 $$\:\mathrm{ln}\left(1-F\right)={K}_{FD}\left(t\right)$$

where *K*_FD_ represents the external film mass transfer coefficient, and *F* denotes the ratio of *q*_t_ to *q*_e_. The slope and intercept of the linear $\:\left(1-F\right)$ plot (Fig. 8d) were used to determine the film diffusion constant (*K*_*FD*_)^53^. Among the kinetic models assessed, the PSOM provided the best description of AB14 dye adsorption onto DRPB-ED. Since the linear plots do not cross the origin, it can be inferred that film diffusion is not the controlling step in the adsorption kinetics. The high correlation coefficient (*R*^2^ > 0.964) obtained for the PSOM further substantiates its reliability in representing the adsorption behavior. Electrostatic interactions between hydrogen ions and negatively charged functional groups on the self-doped activated carbon are likely during the initial adsorption stage, in agreement with PSOM and LIM model predictions. The nitrogen-rich structure of the dye molecules facilitates adsorption onto the positively self-doped DRPB-ED surface, resulting in the formation of a uniform adsorption layer.

**Table 4:** Estimated vs. observed *q*_*e*_ values and PFOM/PSOM rate constants for different AB14 dye and DRPB-ED concentrations.

Parameter			PFOM			PSOM			
DRPB-ED (g/L)	AB14 dye (mg/L)	q_e_ (exp.)	k_1_ × 10^3^	q_e_ (calc.)	*R* ^2^	k_2_ × 10^3^	q_e_ (calc.)	h	*R* ^2^
0.75	50	31.83	0.027	33.52	0.960	0.71	39.37	1093.25	0.996
	75	38.06	0.021	34.20	0.893	0.64	45.45	1318.74	0.974
	100	42.82	0.018	31.46	0.969	0.89	51.28	2329.37	0.993
	125	46.29	0.016	26.88	0.988	0.66	49.75	1627.87	0.984
	150	50.04	0.019	34.16	0.988	0.67	57.47	2215.82	0.994
1.00	50	28.50	0.010	14.30	0.985	1.44	30.30	1320.48	0.964
	75	36.70	0.018	20.08	0.980	1.36	40.00	2168.73	0.992
	100	41.59	0.011	21.77	0.993	0.96	44.84	1935.36	0.976
	125	45.09	0.015	25.51	0.979	0.94	49.50	2298.32	0.988
	150	47.83	0.017	29.45	0.975	0.83	53.19	2334.81	0.988
1.25	50	33.30	0.023	3.78	0.994	14.14	33.67	16025.64	1.000
	75	40.26	0.031	11.86	0.993	5.20	41.49	8960.57	1.000
	100	44.94	0.003	13.85	0.994	4.13	46.51	8936.55	1.000
	125	43.87	0.012	12.28	0.967	2.42	45.05	4904.36	0.995
	150	45.73	0.031	18.30	0.907	3.31	47.39	7423.90	0.999
1.50	50	30.94	0.009	3.23	0.990	10.15	31.06	9784.74	0.999
	75	40.18	0.014	7.78	0.969	4.63	40.82	7716.05	0.999
	100	44.87	0.024	11.71	0.993	4.18	46.30	8968.61	1.000
	125	43.84	0.016	9.99	0.945	3.68	44.84	7401.92	0.998
	150	45.85	0.032	7.87	0.973	8.42	46.73	18382.35	1.000
1.75	50	26.64	0.012	2.67	0.993	13.47	26.81	9680.54	0.999
	75	35.15	0.014	6.70	0.988	5.43	35.71	6920.42	0.999
	100	41.58	0.016	6.71	0.968	5.75	42.19	10235.41	0.999
	125	41.88	0.014	9.86	0.975	3.36	42.92	6184.29	0.998
	150	43.76	0.015	5.92	0.992	6.59	44.25	12903.23	1.000

**Table 5:** Adsorption rate constants of IPDM and FDM models at varying initial concentrations of AB14 dye and DRPB-ED.

DRPB-ED (g/L)	AB14 dye (mg/L)	IPDM			FDM	
		*K*_dif_	*C*	*R* ^2^	*K*_FD_	*R* ^2^
0.75	50	2.312	5.058	0.978	0.027	0.960
	75	2.465	8.002	0.989	0.022	0.893
	100	2.636	10.501	0.995	0.018	0.969
	125	2.541	15.677	0.978	0.016	0.988
	150	3.058	13.849	0.965	0.019	0.988
1.00	50	1.334	10.536	0.948	0.010	0.985
	75	1.692	15.919	0.997	0.017	0.980
	100	2.049	14.752	0.974	0.011	0.993
	125	2.267	16.812	0.996	0.015	0.979
	150	2.501	17.008	0.997	0.017	0.975
1.25	50	0.325	29.591	0.922	0.023	0.994
	75	0.821	31.076	0.914	0.031	0.993
	100	1.064	33.180	0.881	0.030	0.994
	125	1.124	29.177	0.966	0.012	0.967
	150	1.115	32.928	0.961	0.031	0.907
1.50	50	0.310	26.729	0.940	0.009	0.990
	75	0.741	30.953	0.963	0.014	0.969
	100	0.982	33.218	0.924	0.024	0.993
	125	0.851	33.218	0.981	0.016	0.945
	150	0.614	39.153	0.834	0.032	0.973
1.75	50	0.257	23.324	0.979	0.012	0.993
	75	0.644	27.170	0.978	0.014	0.988
	100	0.577	34.370	0.995	0.016	0.968
	125	0.885	30.584	0.983	0.014	0.975
	150	0.542	37.040	0.992	0.015	0.992

### ANN modelling

**Fig. 9:**
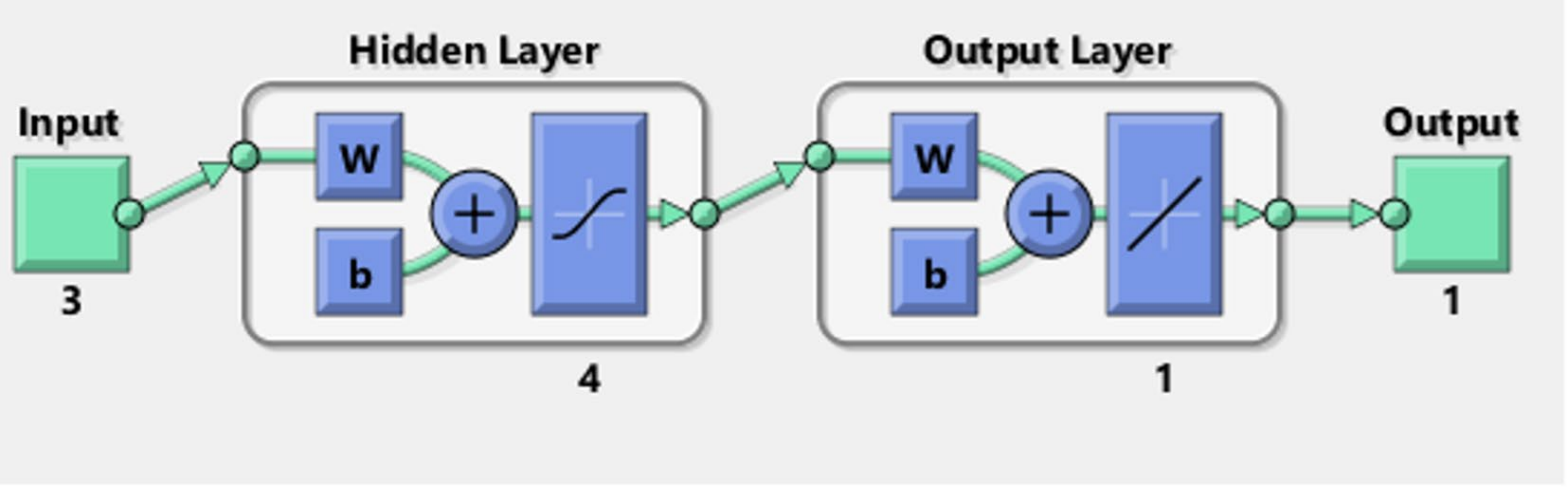
ANN architecture for the elimination of AB14 dye.

**Fig. 10:**
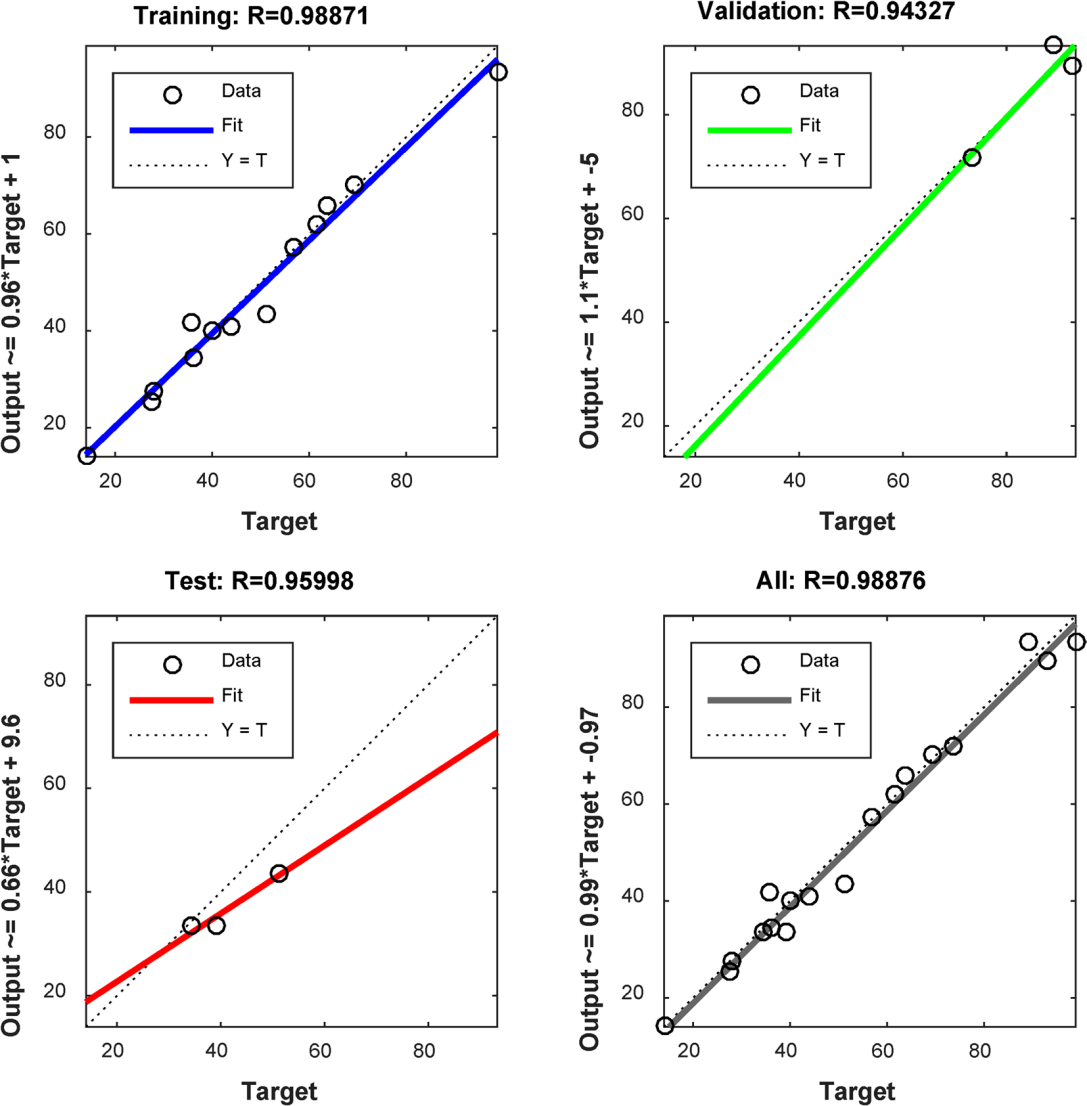
Training, validation, testing, and overall datasets for the LM algorithm.

**Fig. 11:**
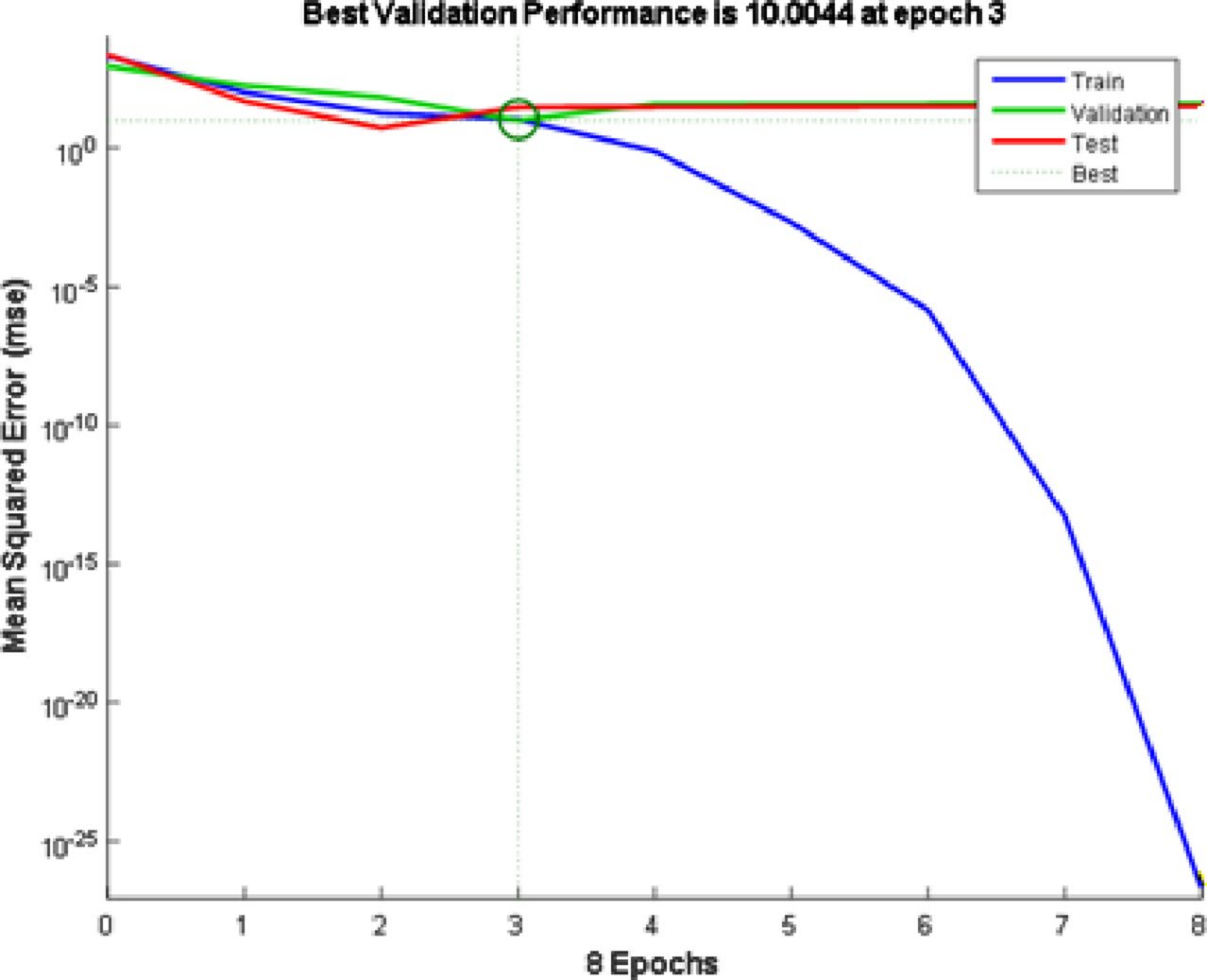
LM algorithm performance.

The ANN model sample is composed of training (70%), testing (15%), and validation (15%) sets, and is trained using the backpropagation algorithm. The selected ANN architecture for modeling AB14 dye removal by DRPB-ED comprised a 3-4-1 structure, with 3 input neurons, 4 hidden neurons, and 1 output neuron (Fig. 9). Figure 10 presents regression plots with *R*^2^ values corresponding to the training, validation, and testing phases of the ANN model. The constructed ANN model’s performance shows that it can predict AB14 dye elimination by DRPB-ED quite well. With high correlation coefficients (*R*^2^ values) of 0.989, 0.943, and 0.960, respectively, the regression plots for the training, validation, and testing datasets demonstrate strong linear correlations between the predicted and experimental values, indicating that the network was successful in learning the nonlinear relationship between the input variables and removal efficiency. With a slope near unity and a modest intercept, the overall regression plot, which incorporates all data subsets, yields an *R*^2^ of 0.989, indicating that the model predictions closely match the target values across the entire dataset. Together, these findings confirm the ANN model’s robustness, reliability, and generalization capacity in accurately reproducing the adsorption process. The overall coefficient of determination (*R*^2^) was 0.989, with a mean squared error (MSE) of 2.27 × 10^–27^. The ANN configuration featured a tan-sigmoid activation for hidden neurons and a purelin activation for the output. Three input parameters—DRPB-ED dosage, contact time, and initial AB14 concentration—were used to predict the output, which was the AB14 dye removal efficiency. Figure 11 illustrates the mean squared error (MSE) in relation to the epoch number for the optimized ANN model. The efficiency and stability of the optimized ANN model for forecasting AB14 dye removal are further confirmed by the change in mean squared error (MSE) with training epoch. During the first few epochs, the MSE curves for the training, validation, and test datasets decline rapidly, demonstrating quick convergence of the backpropagation algorithm and efficient learning of the input-output relationship. This epoch represents the ideal trade-off between underfitting and overfitting, as evidenced by a minimum validation error of roughly 10.00 at epoch 3, which is the best validation performance. After that, further training does not improve and may even slightly worsen the validation error. The model’s good generalization to unobserved data is indicated by the close proximity of the validation and test error profiles, suggesting that the chosen network design and training conditions are suitable and trustworthy for simulating the adsorption process^54^.

Prior to ANN training, all input and output variables were normalized using a min–max scaling approach to the range [–1, 1] to enhance numerical stability and improve convergence of the Levenberg–Marquardt algorithm. To avoid overfitting, the dataset was randomly split into training (70%), validation (15%), and test (15%) sets. Early stopping was applied based on validation performance, with the best validation MSE achieved at epoch 3. The selected 3–4–1 architecture further minimized model complexity. The close agreement between validation and testing R^2^ values confirms the model’s strong generalization ability.

### RSM study

The significance of the selected model and the impact of individual variables on AB14 dye removal were analyzed using ANOVA^55–60^. Tables 1 and 2 present the experimental and projected elimination percentages, along with the ANOVA results. The magnitude of the F-values indicates the extent to which the factors and their interactions influence the response. An F-value exceeding 1 signifies a significant effect of the factor or interaction on the outcome. The percentage of AB 14 clearance was predominantly influenced by the initial AB 14 concentration, as shown in Table 6. The model’s Adeq Precision value of 57.9159 (Table 2) underscores its reliability. Significant factors are identified by p-values below 0.05, and the model’s p-value (< 0.0001) further supports its significance. The difference between adjusted *R*^2^ (0.9896) and predicted *R*^2^ (0.9803) is small (< 0.2), indicating the model’s robustness. The AB14 dye removal percentage can thus be expressed using Eqs. (6) and (7):

6 $$\begin{array}{ccccc}{\rm{Removal }}\% {\rm{ for coded factors }} & = {\rm{49}}.{\rm{94 }} + {\rm{ 21}}.{\rm{32A }} - {\rm{17}}.{\rm{12B }} + {\rm{ 5}}.{\rm{74C}}\\& \quad - {\rm{ 8}}.{\rm{58AB }} - {\rm{ }}0.{\rm{2959AC }} + {\rm{ }}0.0{\rm{58}}0{\rm{BC }}\\& \quad - {\rm{ 1}}.{\rm{66}}{{\rm{A}}^{\rm{2}}} + {\rm{ 4}}.{\rm{21}}{{\rm{B}}^{\rm{2}}} - {\rm{ 2}}.{\rm{91}}{{\rm{C}}^{\rm{2}}}\end{array}$$

7 $$\begin{array}{ccccc}{\rm{Removal }}\% {\rm{ for actual factors }} & = {\rm{ }} - {\rm{21}}.{\rm{62341 }} + {\rm{ 94}}.{\rm{4}}0{\rm{738 }}\left( {{\rm{DRPB}} - {\rm{ED dosage}}} \right){\rm{ }} - {\rm{ }}0.{\rm{25181}}0{\rm{ }}\left( {{\rm{AD14 dye conc}}.} \right){\rm{ }}\\& \quad + {\rm{ }}0.{\rm{251375 }}\left( {{\rm{Time}}} \right){\rm{ }} - {\rm{ }}0.{\rm{343187 }}\left( {{\rm{DRPB}} - {\rm{ED dosage }} \times {\rm{ AD14 dye conc}}.} \right){\rm{ }}\\& \quad - {\rm{ }}0.00{\rm{9864 }}\left( {{\rm{DRPB}} - {\rm{ED dosage }} \times {\rm{ Time}}} \right){\rm{ }} + {\rm{ }}0.0000{\rm{19 }}\left( {{\rm{AD14 dye conc}}.{\rm{ }} \times {\rm{ Time}}} \right){\rm{ }}\\& \quad - {\rm{ 6}}.{\rm{62754 }}{\left( {{\rm{DRPB}} - {\rm{ED dosage}}} \right)^{\rm{2}}} + {\rm{ }}0.00{\rm{1683 }}{\left( {{\rm{AD14 dye conc}}.} \right)^{\rm{2}}} - {\rm{ }}0.000{\rm{8}}0{\rm{7 }}{\left( {{\rm{Time}}} \right)^{\rm{2}}}\end{array}$$

**Table 6:** ANOVA results obtained from the Box–Behnken design for the factors influencing AB14 dye removal.

Source	Sum of squares	df	Mean square	F-value	*p*-value	
Model	12,312.76	9	1368.08	244.93	< 0.0001	Significant
A-DRPB-ED dosage	6263.53	1	6263.53	1121.36	< 0.0001	
B-AD14 Dye Conc.	4622.42	1	4622.42	827.56	< 0.0001	
C-Time	488.37	1	488.37	87.43	< 0.0001	
AB	667.35	1	667.35	119.48	< 0.0001	
AC	0.8254	1	0.825	0.148	0.707	
BC	0.038	1	0.038	0.007	0.935	
A^2^	11.91	1	11.91	2.13	0.166	
B^2^	45.23	1	45.23	8.10	0.013	
C^2^	26.14	1	26.14	4.68	0.048	
Residual	78.20	14	5.59			
Lack of fit	78.20	8	9.77			
Pure error	0.000	6	0.000			
Cor total	12390.96	23				
Std. Dev.	2.36	*R* **²**	0.994			
Mean	48.40	Adjusted *R***²**	0.990			
C.V. %	4.88	Predicted *R***2**	0.980			
Adeq precision	57.9159					

**Fig. 12:**
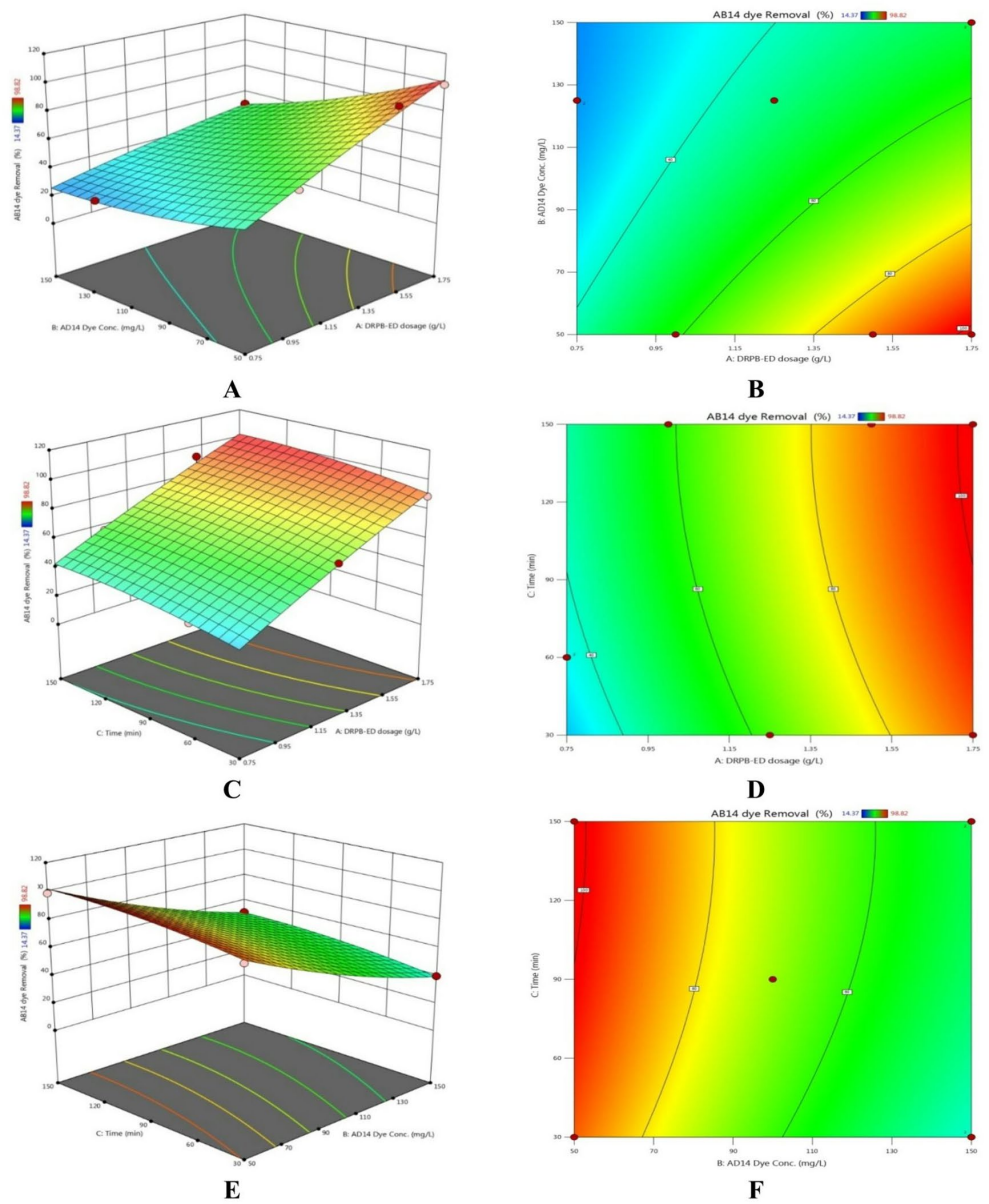
Combined effects of independent variables: (**A**,**B**) DRPB-ED Adsorbent dose and AB 14 dye initial concentration, (**C**,**D**) Adsorbent dose and time, and (**E**,**F**) AB 14 dye initial concentration and time.

**Fig. 13:**
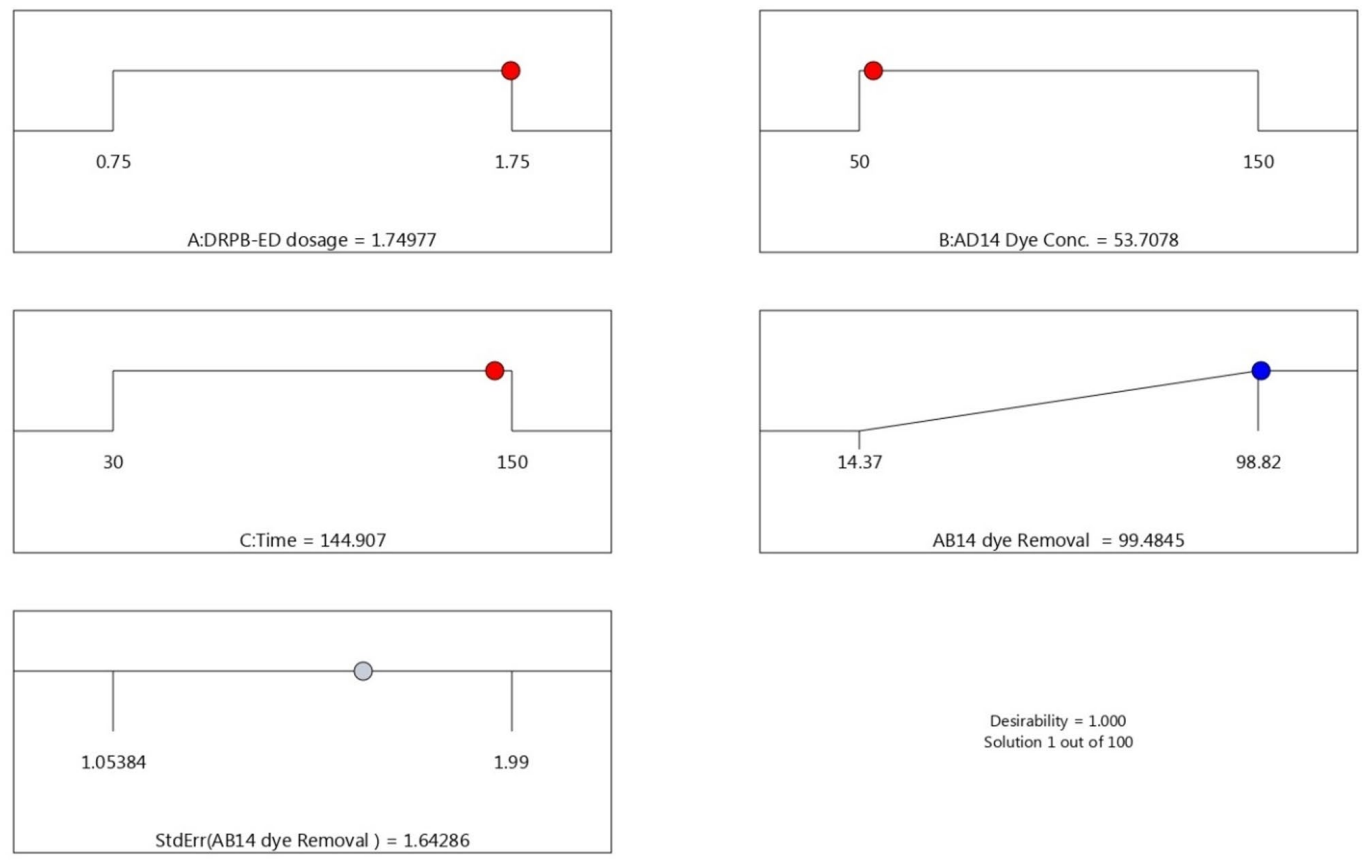
Optimization conditions through DOD settings.

Figure 12 illustrates the cumulative impact of reaction time, adsorbent dosage, and initial AB 14 dye concentration on the percentage of AB 14 dye removal. Optimal removal percentages are achieved through low AB 14 dye concentrations, substantial DRPB-ED adsorbent dosages, and extended reaction periods^56,58,60^. The optimal operating parameters for achieving the highest AB 14 dye elimination percentage were determined statistically, as shown in Fig. 13. Elbager and colleagues^17^ investigated the development of activated carbon obtained from Desert Date Seed Shells (DDSSAC) for methylene blue removal. In their RSM study, they showed that adsorption capacity increased with decreasing adsorbent weight and with increasing pH and methylene blue (MB) concentration in the solution. In this study, the maximum adsorption capacity was 1684 mg/g, obtained at an adsorbent weight of 3 mg, pH of 10, and MB concentration of 300 mg/L. They reported that this was achieved due to increased electrostatic attraction at high pH, efficient use of active sites with low adsorbent weight, and stronger mass transfer driving force at higher dye concentrations^17^.

The RSM model showed excellent statistical performance (*R*^2^ = 0.994, Predicted *R*^2^ = 0.980, *p* < 0.0001), while the ANN model achieved an overall R² of 0.989 with strong validation (*R*^2^ = 0.943) and testing (*R*^2^ = 0.960) agreement, confirming good generalization. The close similarity in predictive accuracy demonstrates strong consistency between RSM-optimized and ANN-predicted results. While RSM provides an explicit quadratic equation suitable for statistical interpretation and optimization, ANN offers greater flexibility in capturing nonlinear adsorption behavior. This complementary agreement strengthens the robustness and reliability of the developed predictive framework, consistent with recent benchmark studies^17^.

### Comparison with the result documented in the literature

To assess the efficiency of different adsorbents for azo dye removal, a comparison with literature data was carried out, highlighting the performance of DRPB-ED. The corresponding adsorption capacities (mg/g) are summarized in Table 7. The results clearly indicate that DRPB-ED exhibits excellent removal efficiency for AB14 dye, confirming its strong potential as a competitive adsorbent.

**Table 7:** Comparison of DRPB-ED adsorption capacities for AB14 dye.

Adsorbent	Dye	Q _m_ (mg/g)	Ref.
Fennel seeds	MR	135.00	^61^
Biogas plant waste	MR	113.00	^62^
Marigold	MR	102.43	^63^
Refused tea waste activated carbon	AY36	71.97	^64^
AC from peanut shells	AY36	66.70	^65^
Green nanoceria (GN) and GN–NH_2_ (AGN) from Prosopis julifora leaves extract	AY36	26.95	^66^
Mandarin-CO-TETA derived from mandarin peels	AB14	416.67	^42^
Self-nitrogen-doped porous activated carbon	AB14	107.50	^18^
DRPB-ED	AB14	60.61	This Study

### Regeneration study

**Fig. 14:**
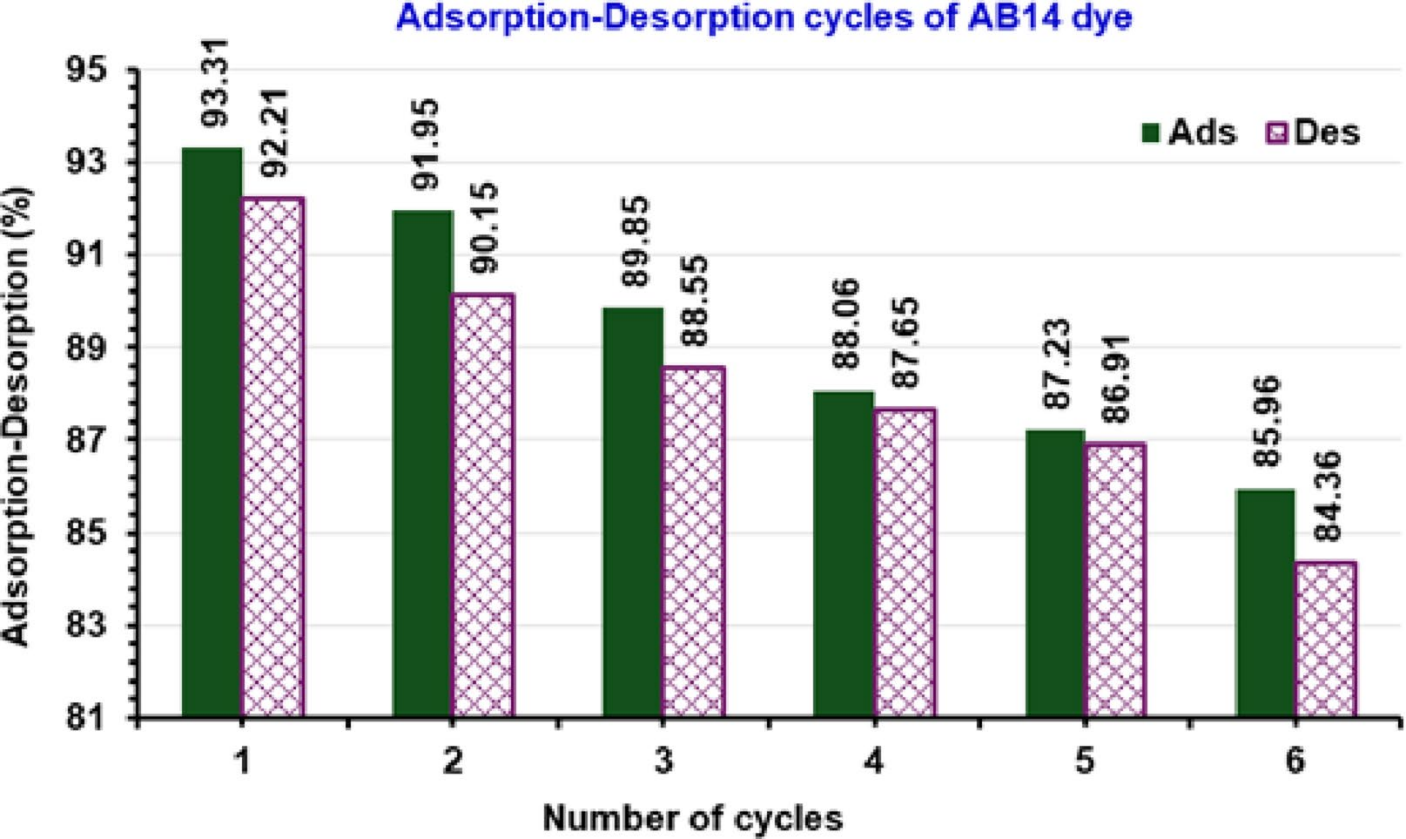
Regeneration study of AB14 by DRPB-ED adsorbent using dye *C*_0_ (50 mg/g) and 1.75 g/L DRPB-ED dose at room temperature.

Desorption experiments were conducted to evaluate the regeneration efficiency and reusability of the DRPB-ED adsorbent for AB14 dye removal. A 0.1 M NaOH solution was used as the desorbing agent, and the concentration of the eluted dye was measured to determine the desorption efficiency. After each cycle, the spent DRPB-ED was reactivated using 0.1 M HCl. As shown in Fig. 14, the percentage of dye desorption gradually decreased with increasing regeneration cycles. The regenerated DRPB-ED was subjected to six successive adsorption–desorption cycles. The adsorption and desorption performances remained nearly constant throughout the cycles; however, after six cycles, the adsorption capacity decreased by approximately 7.35% for AB14 dye (Fig. 14). These results indicate that DRPB-ED is a promising and durable adsorbent for the removal of AB14 dye from aqueous solutions^67,68^. Furthermore, a thermodynamic investigation of AB14 dye removal at different temperatures would provide valuable insight into the feasibility and practical applicability of the DRPB-ED adsorbent^69,70^. In addition, a comprehensive techno-economic analysis is essential to evaluate the economic viability of the proposed adsorbent for large-scale applications^71^.

## Conclusion

This study highlights the potential of ethylenediamine-modified DRPB (DRPB-ED) as an efficient, sustainable, and economically viable adsorbent for removing AB14 dye from aqueous solutions. The modification notably enhanced the adsorptive performance compared to unmodified biochar, offering a cost-effective alternative to conventional activated carbon. Experimental results demonstrated that optimal dye removal occurred under acidic conditions (pH 1.5) with an adsorbent dosage of 1.75 g/L, achieving a high removal efficiency of 93.23%. The equilibrium data were best represented by the Langmuir isotherm, signifying monolayer adsorption on a uniform surface with a maximum adsorption capacity of 60.61 mg/g. The adsorption kinetics were best described by the pseudo-second-order model, indicating that chemisorption, predominantly via hydrogen bonding and electrostatic forces, was the rate-determining step. Equilibrium was reached within 150 min, confirming the rapid and effective adsorption behavior of DRPB-ED.

Overall, the findings demonstrate that DRPB-ED exhibits high adsorption capacity, fast kinetics, and favorable surface properties, making it a promising adsorbent for industrial wastewater treatment. Future studies should focus on regeneration and reuse to evaluate their long-term economic feasibility, as well as on testing their performance against various organic and inorganic contaminants. The results advance sustainable adsorbent materials and provide valuable insights for the development of eco-friendly wastewater treatment technologies. RSM was employed to optimize the parameters, revealing that the application of 1.75 g of the DRPB-ED adsorbent and 53.71 ppm of AB 14 solution for 144.9 min resulted in the maximum degradation of 99.48%.

The results of this study indicate that DRPB-ED is a promising adsorbent for the removal of Acid Brown 14 under controlled laboratory conditions. However, conclusions regarding its industrial scalability and long-term sustainability should be considered preliminary. Further investigations, including adsorbent regeneration, long-term stability, performance in real wastewater matrices, and techno-economic assessment, are required to fully evaluate its feasibility for practical and large-scale wastewater treatment applications.

## Data Availability

The datasets used in this study are available for review upon request from the corresponding author.
